# State-aware adaptive multimodal fusion for ultra-short-term solar irradiance forecasting using sky cloud images

**DOI:** 10.1371/journal.pone.0362193

**Published:** 2026-11-03

**Authors:** Houhang Zhao, Aiyi Song, Yu Jin, Haoxiang Yu

**Affiliations:** 1 School of Statistics, Dongbei University of Finance and Economics, Dalian, China; 2 School of Health and Medical Technology, Dalian Neusoft University of Information, Dalian, China; 3 Academy for Educational Development and Innovation, The Education University of Hong Kong, Hong Kong, China; Dr Shakuntala Misra National Rehabilitation University, INDIA

## Abstract

As renewable energy penetration continues to increase, accurate ultra-short-term solar irradiance forecasting is essential for improving energy utilization and supporting power system operation. However, irradiance variations are jointly influenced by historical trends and rapid cloud changes, making traditional single-modal methods insufficient for capturing complex dynamics under varying weather conditions. This paper proposes a multimodal solar irradiance forecasting framework based on the collaborative representation of sky cloud images and numerical sequences. The framework introduces a heterogeneous modal state encoding mechanism and a state-aware adaptive selective attention fusion mechanism to enhance interactions between visual weather information and historical physical patterns. Specifically, the numerical modality captures irradiance trends and temporal correlations through dependency modeling, while the image modality extracts cloud structures and evolution features through spatiotemporal representation. The proposed fusion mechanism dynamically adjusts modality contributions according to forecasting conditions, enabling effective integration of complementary information. Experimental results demonstrate that the proposed framework outperforms benchmark models in forecasting accuracy and robustness, validating the effectiveness of combining visual cloud information with numerical evolution patterns for ultra-short-term solar irradiance forecasting.

## 1. Introduction

With the ongoing advancement of the “dual carbon” goals and the accelerated development of a new power system, the share of solar photovoltaic (PV) power in the energy mix continues to rise. Compared with traditional controllable power sources, solar irradiance exhibits significant fluctuations and uncertainty; its variations are influenced not only by regular factors such as diurnal cycles and seasonal changes but also by random factors such as sudden changes in local weather [1]. Particularly in ultra-short-term forecasting scenarios, the rapid formation or dissipation of clouds over observation stations or solar power plants can cause sharp increases or decreases in solar irradiance, which in turn affect estimates of solar power output and pose challenges to renewable energy integration and the safe and stable operation of the power system [2]. Therefore, developing an ultra-short-term solar irradiance prediction model capable of accurately detecting cloud-induced fluctuations and rapidly responding to changes in irradiance holds significant theoretical and engineering value. In recent years, with the rapid penetration of wind and solar energy into energy systems, artificial intelligence techniques have been increasingly applied to energy efficiency optimization and intelligent energy management. Recent studies have demonstrated the potential of AI-based approaches in improving energy utilization efficiency and supporting intelligent energy system operation [3]. Meanwhile, research on renewable energy forecasting based on time-series feature engineering, ensemble learning, and artificial intelligence methods has continued to advance, further driving the development and optimization of photovoltaic forecasting models under complex conditions [4,5].

Most existing methods for predicting solar irradiance rely on structured numerical data, such as historical irradiance records or numerical weather forecasts, and use these continuous numerical variables to build predictive models. Given that solar irradiance time series are inherently highly stochastic and exhibit distinct seasonal and weather-related fluctuations, researchers have conducted extensive studies using various approaches, including statistical methods, machine learning, and deep learning [6,7]. Bacher et al. [8] proposed an online short-term solar power forecasting method that combines numerical weather prediction with recursive least squares to forecast the short-term solar power output of a rooftop photovoltaic system in Denmark. Pedro and Coimbra [9] systematically compared various solar power forecasting techniques without exogenous variables, demonstrating that forecasts based solely on historical solar irradiance or power time series can achieve a certain level of accuracy under relatively stable weather conditions. Sharadga et al. [10] further compared the performance of statistical and artificial intelligence models in solar power forecasting, validating the feasibility of modeling based on historical time series. Although these methods can effectively capture the continuous patterns of solar irradiance, historical irradiance values primarily reflect past solar radiation conditions and thus fail to fully characterize leading indicators such as sudden local weather changes and future irradiance variations. Consequently, under complex weather conditions, these methods are prone to prediction lags or amplified errors [11].

To address the limitations of relying solely on historical irradiance data, subsequent studies incorporated exogenous meteorological variables and numerical weather forecast information to characterize the dynamic behavior of solar irradiance under varying climatic conditions. For example, Mellit et al. [12] compared deep learning networks, including LSTM, GRU, and one-dimensional CNNs, for short-term solar irradiance forecasting; du Plessis et al. [13] investigated the ability of deep learning models to capture operational behavior in utility-scale photovoltaic systems; and Transformer-based architectures and attention mechanisms have been introduced to enhance the representation of weather categories and multiscale features [14]. By expanding input variables, these methods provide models with additional weather-related information, improving prediction accuracy under different seasons and weather conditions. However, historical irradiance time series still struggle to provide timely information on cloud shading due to the rapid evolution of cloud cover. Moreover, meteorological observations and numerical weather forecasts are limited by spatial resolution and update frequency, making it difficult to accurately characterize local cloud evolution [15,16]. Therefore, numerical models alone remain insufficient for ultra-short-term forecasting under local occlusion and sudden weather variations. With the development of multimodal deep learning, image information has been further incorporated to improve spatial perception of cloud fields [17,18].

To overcome the spatial perception limitations of numerical models, researchers have introduced satellite imagery to characterize cloud field distributions [19,20]. Deep learning methods, including convolutional neural networks, residual networks, and attention mechanisms, enable satellite images to extract cloud spatial distributions and evolution patterns. In addition to image-based information, recent studies have also incorporated multiple environmental variables into data-driven solar forecasting models to characterize the influence of atmospheric conditions on solar energy variations [21]. Combined with irradiance or meteorological variables, these features improve the ability of forecasting models to capture cloud-induced irradiance changes [22]. Si et al. [23] integrated cloud motion prediction with an XGBoost model considering solar position, while Xie et al. [24] utilized satellite image texture features and transfer learning for regional solar radiation forecasting. Compared with numerical models, satellite imagery provides more intuitive cloud information; however, its large coverage area and limited update frequency restrict its application in minute-level ultra-short-term forecasting of local cloud variations [20,25]. In contrast, ground-based sky images provide higher spatiotemporal resolution and can directly capture local cloud cover and solar occlusion, making them suitable for minute-to-hour-scale cloud dynamics analysis [26]. Kamadinata et al. [27] used sky images and artificial neural networks for solar irradiance prediction, while Feng et al. [28] proposed a CNN-based model using sky image sequences for minute-to-hourly solar energy forecasting. These studies demonstrate that sky images provide valuable visual weather information, particularly under complex conditions such as overcast skies and rapidly moving clouds [29].

However, existing studies based on satellite imagery or sky images still mainly rely on a single image modality for solar irradiance forecasting. Although images provide rich visual information on weather conditions, they cannot directly reflect historical irradiance variations. Meanwhile, image quality and insufficient information in individual frames may affect feature stability, causing models to overlook temporal evolution patterns, especially under complex weather conditions. Paletta et al. [30] showed that although spatiotemporal image features improve short-term forecasting performance, some models remain limited in detecting abrupt events, such as cloud-induced solar occlusion, in advance. Therefore, image-based and numerical time-series approaches exhibit natural complementarity in solar irradiance forecasting: images provide external visual information on cloud evolution and occlusion events, whereas numerical sequences provide historical irradiance trends and temporal constraints. Their fusion can improve the characterization of irradiance variations under complex weather conditions [31].

Regarding the fusion of image and numerical modalities, existing methods can generally be classified into output-level fusion, feature-level fusion, and deep learning-based joint fusion [32]. Some studies separately construct models using image and numerical information and then combine predictions through weighted averaging or ensemble learning. However, such post-fusion strategies only integrate model outputs and fail to exploit cross-modal interactions during feature learning, limiting their ability to capture correlations between cloud variations and irradiance responses [33]. Another approach extracts image features, such as weather categories, and incorporates them as external inputs into forecasting models. For example, Xie, et al. [34] performed short-term solar irradiance forecasting by extracting features from sky images and combining them with an SSA-BiLSTM; Ajith and Martínez-Ramón [26] fused infrared cloud images with irradiance data for microscale solar radiation prediction. Although these methods improve the utilization of visual information, compressing images into limited features may result in the loss of cloud spatial structures and motion characteristics.

In recent years, with the advancement of deep multimodal learning, solar forecasting has gradually evolved from simple feature combination toward more adaptive and interactive fusion mechanisms [32]. Feature concatenation provides a straightforward way to integrate high-level representations from different modalities; however, this relatively static combination strategy generally assumes that different modalities contribute with fixed or nearly fixed importance, making it difficult to adaptively adjust their contributions under changing forecasting conditions [35]. To enhance modal selection capabilities, Zhang, et al. [33] proposed an advanced multimodal fusion method based on sky images and meteorological data, which introduced a gate and Transformer mechanism to improve multimodal feature integration. Nevertheless, such gating decisions are mainly derived from latent feature representations and therefore do not fully characterize variations in modality reliability across different forecasting states. In addition, some studies have begun to shift their research focus from modality weighting to cross-modal interaction, establishing correlations between visual and temporal representations to enhance cross-modal information transfer and joint feature learning [36]. However, such methods primarily focus on feature matching and information transfer between modalities, aiming to improve cross-modal representation capabilities. They do not explicitly consider that the reliability and relative importance of image and numerical information may vary under different weather conditions, cloud states, and irradiance variation patterns. To further incorporate weather-state information into the fusion process, Wang et al. [37] developed an ultra-short-term solar irradiance forecasting method based on cloud-type guidance and multimodal fusion, using cloud-type-guided attention to enhance cloud-information representation and improve multimodal feature integration. This strategy enables the fusion process to account more explicitly for cloud-state characteristics; however, its main focus remains the enhancement and integration of cloud-related features, while the relative reliability of image and numerical modalities for individual forecasting samples is not explicitly evaluated or dynamically adjusted according to different weather and irradiance states. Therefore, developing effective deep multimodal fusion mechanisms that fully exploit the complementary characteristics of image and numerical modalities remains essential for improving ultra-short-term solar irradiance forecasting performance.

Given the limitations of existing methods in terms of multimodal information interaction mechanisms and adaptability to complex weather scenarios, this paper proposes TSGAN (Transformer Selective Gated Attention Network), a Transformer-based, state-aware adaptive fusion forecasting framework. By synergistically fusing visual information from sky cloud images with structured numerical sequence data, this framework enhances the model’s ability to capture complex dynamic changes in ultra-short-term solar irradiance. The framework first constructs a Unified State Encoder (USE) with heterogeneous representation paths tailored to the data characteristics of different modalities. For the numerical modality, a Transformer encoding structure is utilized to model deep temporal relationships among historical irradiance and meteorological variables, and a global dependency capture mechanism is employed to extract dynamic evolutionary patterns and latent correlation features associated with irradiance changes. For the image modality, a visual encoding branch integrating spatial perception and temporal evolution is constructed. A convolutional feature extraction mechanism captures local spatial structures in sky cloud patterns, while a temporal modeling mechanism further characterizes dynamic information such as cloud movement and visual state changes, thereby forming a visual state representation with spatiotemporal consistency. After obtaining high-level state representations from different modalities, this paper further designs a selective gated attention network as the core cross-modal fusion module. By introducing a modality-state-aware mechanism, the network dynamically adjusts the relative contributions of the image and numerical modalities across different forecasting scenarios. Unlike existing gated fusion methods, which primarily rely on latent space feature representations to generate modal weights, TSGAN first performs joint modeling of different modal states, using both the evolutionary information of numerical sequences and the visual state of the sky-cloud map as the basis for gating decisions, and generates dynamic modal selection weights based on the environmental state of the current predicted sample. Furthermore, unlike Cross-Attention methods, which primarily focus on feature correlations and information exchange between different modalities, TSGAN further accounts for differences in the effectiveness of different modalities under current forecast conditions, achieving adaptive adjustment of modal contributions through a state-driven information filtering mechanism. This mechanism avoids the static assumptions about modal importance inherent in traditional feature concatenation or fixed-weight fusion methods, enabling the model to dynamically adjust the participation levels of image and numerical modalities based on weather conditions, thereby improving the model’s responsiveness to irradiance changes and prediction stability under complex weather conditions. The main contributions of this paper are as follows:

(1) We propose TSGAN, a multimodal solar irradiance forecasting framework based on a Transformer and a selective gated attention network. By constructing a collaborative representation system for sky cloud images and numerical time series, TSGAN enables the joint modeling of visual weather information and historical physical patterns, effectively addressing the information gaps inherent in single-modality approaches regarding spatial perception or temporal evolution.
(2) We construct a dual-branch state encoding structure tailored to heterogeneous inputs. The Transformer temporal encoder extracts long-term dependencies and dynamic patterns from the numerical modality, while a visual encoding mechanism captures cloud structures and motion characteristics from sky cloud images, thereby yielding complementary multimodal state representations.
(3) We propose a selective gated attention network that transforms the multimodal fusion process from static feature combination into dynamic information selection based on sample-state awareness. This network can adaptively adjust the fusion weights of image and numerical features according to changes in modality reliability under different weather conditions, enabling more refined cross-modal information interaction.

## 2. Methodology

To validate the effectiveness and superiority of the proposed method, this study constructs an overall research framework, as shown in Fig 1. The framework primarily consists of four steps: (1) multimodal state encoding and feature alignment; (2) state-aware adaptive modality fusion; (3) multimodal fusion forecasting; and (4) model performance evaluation.

**Fig 1:**
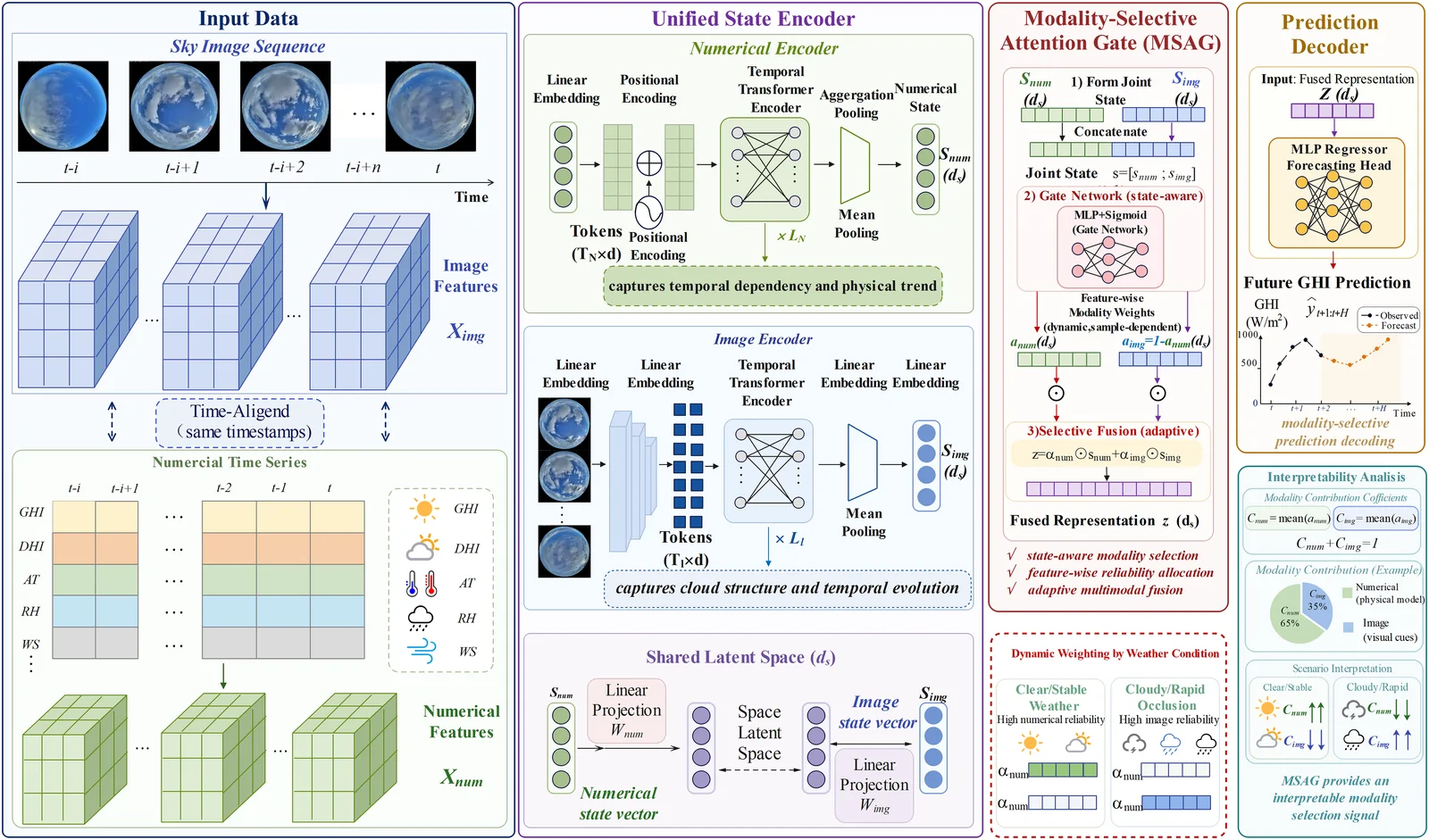
Forecasting framework. The sky images shown in the input-data panel were adapted from the Open Energy Data Initiative (OEDI) dataset and are licensed under the Creative Commons Attribution 4.0 International License (CC BY 4.0).

The TSGAN proposed in this paper is designed for ultra-short-term GHI forecasting tasks, and its overall architecture consists of a unified state encoder and a modality-selective predictive decoder. The unified state encoder extracts heterogeneous state representations from numerical sequences and sky images, while the modality-selective predictive decoder employs a modality-selective attention gating mechanism as its core fusion module to dynamically filter and adaptively fuse the two types of representations, ultimately outputting GHI forecasts for future time steps. Unlike traditional multimodal models, which directly concatenate features from different modalities and feed them into the prediction layer, the core idea of TSGAN is to explicitly model the multimodal fusion process as a dynamic decision-making process involving “modality-state identification—modality-importance allocation—complementary information fusion,” enabling the model to autonomously determine the relative contributions of the numerical and image modalities to the forecast based on the current sample state. Specifically, the numerical modality consists of historical GHI, DNI, DHI, and meteorological variables, primarily describing the historical inertia and short-term evolution trends of irradiance; the image modality consists of continuous sky cloud images, primarily providing visual priors such as cloud-cover information and changes in sky brightness. Although the two modalities are complementary, their relative importance varies across different weather scenarios. Therefore, this paper does not adopt a fusion strategy based on fixed weights or simple concatenation; instead, it incorporates a modality-selective attention gating mechanism as the core design of TSGAN, enabling the model to adaptively determine the effective contributions of the two modalities at both the sample and feature-dimension levels.

### 2.1 Unified state encoder

The goal of a unified state encoder is to map numerical sequences and sky images onto the same latent space, providing comparable high-level representations for subsequent modal selection and fusion. Given a history observation window of length L, the numerical modal input is denoted as $X^{num}\epsilon \;R^{L\times F}$, where F is the dimension of the numerical variables; the time-aligned sequence of sky images is denoted as $X^{img}\epsilon \;R^{L\times C\times H\times W}$. The prediction process of TSGAN can be formalized as:


$$\begin{array}{c} {\hat{y}}_{t+\tau }=F_{\theta }\left(X^{num},X^{img}\right) \end{array}$$ (1)

Here, ${\hat{y}}_{t+\tau }$ represents the GHI prediction values for the next τ steps, and $F_{\theta }$ is a nonlinear mapping function composed of a state encoder, a gated fusion module, and a prediction decoder.

For numerical modalities, this paper employs a temporal Transformer encoder to extract dynamic dependencies among historical physical variables. First, the raw numerical sequences are mapped to a unified latent space, with positional encoding added to preserve temporal order; subsequently, the Transformer encoder models global dependencies across different time steps to obtain contextual representations of the numerical modalities:


$$\begin{array}{c} Z_{num}^{\text{0}}=X^{num}W_{n}+b_{n}+PE_{num} \end{array}$$ (2)

$$\begin{array}{c} Z_{num}=TransformerEncoder_{num}\left(Z_{num}^{\text{0}}\right) \end{array}$$ (3)

$$\begin{array}{c} h_{num}=\text{Pool(}Z_{num}) \end{array}$$ (4)

Here, ${𝒵}_{num}$ represents the high-level temporal representation of the numerical sequence, and $h_{num}\in {\mathbb{R}}^{d}$ is the aggregated numerical modal state vector. This branch captures the nonlinear relationship between GHI and meteorological variables while preserving the overall trend of change within the historical window.

For the image modality, this paper adopts an encoding architecture that combines a CNN with a temporal Transformer. A lightweight CNN encoder is first employed to extract spatial features from each sky image, including information related to solar occlusion regions, cloud boundaries, and local texture variations. Specifically, the CNN encoder consists of four convolutional blocks. Each block uses a 3×3 convolution with a stride of 2, followed by Batch Normalization and a ReLU activation function. The channel dimensions of the four convolutional layers are set to 32, 64, 128, and 256, respectively, corresponding to a progressive transformation from 3 input channels to 32, 64, 128, and 256 feature channels. After the four convolutional blocks, Adaptive Average Pooling is applied to compress the spatial feature maps into a fixed length representation, which is subsequently projected into a 128-dimensional visual feature space through a fully connected layer to form the visual token of each image frame. The visual features of consecutive image frames are then organized into a temporally ordered sequence of visual tokens and fed into the temporal Transformer to model the dynamic evolution of the cloud sequence. The Transformer for the image modality uses the same hidden dimension as the numerical modality ($d_{model}=128$), with four attention heads and one Transformer Encoder layer to capture temporal dependencies among consecutive sky images:


$$\begin{array}{c} e_{t}^{img}=f_{cnn}\left(I_{t}\right),\;t=1,2,...,L \end{array}$$ (5)

$$\begin{array}{c} Z_{img}^{\text{0}}=[e_{\text{1}}^{img},e_{\text{2}}^{img},...,e_{L}^{img}]+PE_{img} \end{array}$$ (6)

$$\begin{array}{c} Z_{img}=TransformerEncoder_{img}\left(Z_{img}^{\text{0}}\right) \end{array}$$ (7)

$$\begin{array}{c} h_{img}=\text{Pool(}Z_{img}) \end{array}$$ (8)

Here, $h_{img}$ represents the image modal state vector. This branch not only describes the spatial structure of a single-frame cloud image but also captures the continuous trends in cloud cover within the historical window, thereby providing visual prior information for short-term GHI fluctuations.

### 2.2 State-aware adaptive fusion mechanism

After obtaining the numerical state vector $h_{num}$ and the image state vector $h_{img}$, the model further enables dynamic interaction between the two types of modal information through an adaptive attention fusion module. Since the effectiveness of image and numerical information varies under different weather conditions, this paper constructs a state-aware weight-allocation mechanism that adaptively adjusts the contributions of features from different modalities based on the joint state of the current sample, enabling the model to fully leverage the historical evolution patterns in numerical time series and the visual prior information in sky cloud images. This module first jointly models the states of the two modalities to generate the corresponding modality-selection weights and then dynamically filters and fuses different feature dimensions based on these weights. Compared with fixed fusion strategies, this method can flexibly adjust modality dependencies in response to changing weather conditions, effectively utilizing historical trends under stable weather conditions while enhancing the role of visual information in scenarios such as rapid cloud movement or solar occlusion, thereby obtaining a more discriminative fused state representation.

Specifically, the state vectors of the two modalities are first concatenated to form$r=[h_{num};h_{img}]$ joint state representation. The joint state vector r contains both the numerical modality’s representation of historical physical trends and the image modality’s representation of the visual state of the sky; therefore, it can be regarded as a multimodal state summary of the current forecasting sample. Next, modality-selection weights are generated using a lightweight attention-gating network. In a two-modality scenario, the sigmoid gate can be viewed as a form of binary soft-attention allocation:


$$\begin{array}{c} s=W_{g}r+b_{g} \end{array}$$ (9)

$$\begin{array}{c} g=\sigma \left(s\right),\;g\in (0,1{)}^{d} \end{array}$$ (10)

$$\begin{array}{c} a_{num}=g,\;a_{img}=1-g \end{array}$$ (11)

Here, s represents the modal selection score derived from the joint state mapping, g denotes the attention weight of the numerical modality on each latent feature dimension, and 1−g denotes the attention weight of the image modality on the corresponding dimension. Since g is determined by the current sample’s joint state r, this weight is not a fixed parameter but rather a modality-reliability response that changes dynamically with the input sample. If the historical numerical sequence in a given sample exhibits a stable trend, the gating network can increase the weight of the numerical features; if the sky image shows obvious cloud cover or sudden changes in brightness, the gating network can enhance the contribution of the image features in the relevant dimensions. Ultimately, the model obtains a fused representation through dimension-wise weighting and feeds it into the forecasting decoder to predict solar irradiance at future time steps:


$$\begin{array}{c} h_{\text{fuse}}=a_{num}⊙h_{num}+a_{img}⊙h_{img}=g⊙h_{num}+(1-g)⊙h_{img} \end{array}$$ (12)

$$\begin{array}{c} {\hat{y}}_{t+\tau }=f_{dec}\left(h_{\text{fuse}}\right) \end{array}$$ (13)

Here, ⊙ denotes element-wise multiplication, and $f_{dec}(\cdot )$ represents the prediction decoder. From a technical perspective, the distinction of the proposed mechanism lies in how the modality weights are generated and applied. The gate $g\in (0,1{)}^{d}$ is directly conditioned on the joint multimodal state $r=\left[h_{num};h_{img}\right]$, rather than being a fixed scalar or a global modality coefficient. Moreover, the two modality weights are constrained to be complementary at each latent feature dimension, i.e., $a_{num}=g$ and $a_{img}\text{=1-}g$. Therefore, the fused representation is obtained through feature-wise competitive weighting rather than simple feature concatenation or pairwise attention-based feature interaction. Accordingly, $g\in (0,1{)}^{d}$ is a weight vector at the feature dimension level; therefore, TSGAN does not merely decide “whether to choose numerical values or images as a whole,” but performs fine-grained information filtering on each hidden feature dimension, enabling the model to simultaneously preserve the temporal evolution patterns in the numerical modality and the cloud variation information in the image modality. To further explain the model’s overall dependence on different modalities at the sample level, we can define a modal contribution coefficient:


$$\begin{array}{c} a_{num}=\frac{\text{1}}{d}\sum_{k=1}^{d}g_{k}\text{,}a_{img}=1-a_{num} \end{array}$$ (14)

When $a_{num}$ is large, it indicates that the model considers the current sample to be more dependent on the physical trends in the historical data sequence; when $a_{img}$ is large, it indicates that the model considers the visual information of cloud layers in the sky images to be more critical for the prediction. Thus, TSGAN not only performs feature fusion but also provides an interpretable modal selection signal that can be used to analyze how the model’s reliance on the two modalities changes under different weather conditions. The fused representation is subsequently fed into the prediction decoder to generate the final irradiance forecast. The prediction decoder consists of three fully connected transformations with dimensions of 128−128, 128−64, and 64−1, respectively. GELU activation is applied after the first two linear transformations, and a dropout layer is applied after the first hidden layer. The entire model is trained end-to-end using the mean squared error (MSE) loss. Since the target GHI values are standardized before model training, the training objective is defined in the standardized target space as:


$$\begin{array}{c} L_{MSE}=\frac{\text{1}}{N}\sum_{i=1}^{N}({\tilde{y}}_{i}-{\hat{\tilde{y}}}_{i}{)}^{\text{2}} \end{array}$$ (15)

where N denotes the number of samples in a mini-batch, while ${\tilde{y}}_{i}$ and ${\hat{\tilde{y}}}_{i}$ represent the standardized observed and predicted irradiance values, respectively. The gradients of this loss are backpropagated through the prediction decoder, the modality selective attention gating module, and both modality encoders to jointly optimize all trainable parameters.

### 2.3 Evaluation metrics and statistical significance test

To comprehensively evaluate the performance of the proposed model in the solar irradiance forecasting task, this paper uses mean absolute error (MAE), symmetric mean absolute percentage error (SMAPE), normalized root mean square error (NRMSE), and the coefficient of determination ($R^{\text{2}}$) as evaluation metrics. Among these, MAE measures the average absolute deviation between predicted and observed values and provides an intuitive measure of the model’s overall forecasting error; SMAPE evaluates the deviation between predicted and observed values in terms of relative error, thereby mitigating the impact of differences in data scale across varying irradiance levels; NRMSE emphasizes larger errors and is therefore better suited to evaluating the model’s ability to capture irradiance fluctuations under sudden weather changes, such as cloud cover; and $R^{\text{2}}$ measures how well the predicted values fit the observed trend, reflecting the model’s ability to capture the overall evolution of solar irradiance. The formulas for these metrics are as follows:


$$\begin{array}{c} MAE=\frac{\text{1}}{N}\sum_{i=1}^{N}\left|y_{i}-{\hat{y}}_{i}\right| \end{array}$$ (16)

$$\begin{array}{c} SMAPE=\frac{100\%}{N}\sum \frac{\left|y_{i}-{\hat{y}}_{i}\right|}{(\left|y_{i}\right|+\left|{\hat{y}}_{i}\right|)/2} \end{array}$$ (17)

$$\begin{array}{c} NRMSE=\frac{\sqrt{\frac{\text{1}}{N}\sum_{i=1}^{N}(y_{i}-{\hat{y}}_{i}{)}^{\text{2}}}}{y_{max}-y_{min}} \end{array}$$ (18)

$$\begin{array}{c} R^{\text{2}}=1-\frac{\sum_{i=1}^{N}(y_{i}-{\hat{y}}_{i}{)}^{\text{2}}}{\sum_{i=1}^{N}(y_{i}-\overset{―}{y}{)}^{\text{2}}} \end{array}$$ (19)

To examine the statistical significance of the forecasting performance differences between different models, this study employs the Diebold-Mariano (DM) test to perform pairwise comparisons between TSGAN and each baseline model. For each forecasting horizon, the absolute forecasting errors of TSGAN and the corresponding baseline model are calculated on the test set, and the loss differential series is constructed as follows:


$$\begin{array}{c} d_{i}=\left|y_{i}-{\hat{y}}_{TSGAN,i}\right|-\left|y_{i}-{\hat{y}}_{model,i}\right| \end{array}$$ (20)

$$\begin{array}{c} \overset{―}{d}=\frac{\text{1}}{N}\sum_{i=1}^{N}d_{i} \end{array}$$ (21)

$$\begin{array}{c} DM=\frac{\overset{―}{d}}{\sqrt{\frac{\text{1}}{N}\hat{V}\left(d\right)}} \end{array}$$ (22)

where $y_{i}$ denotes the observed irradiance value of the i-th test sample, while ${\hat{y}}_{TSGAN,i}$ and ${\hat{y}}_{model,i}$ represent the forecasts produced by TSGAN and the corresponding baseline model, respectively. $d_{i}$ denotes the loss differential between the two models for the i-th sample, N is the number of test samples, $\overset{―}{d}$ is the mean of the loss differential series, and $\hat{V}\left(d\right)$ denotes the long-run variance estimate of the loss differential series. Since forecast errors from overlapping multi-step forecasts may exhibit serial dependence, the Newey–West heteroskedasticity and autocorrelation consistent estimator with Bartlett weights is employed:


$$\begin{array}{c} \hat{V}\left(d\right)={\hat{\gamma }}_{\text{0}}+2\sum_{k=1}^{h-1}(1-\frac{k}{h}){\hat{\gamma }}_{k} \end{array}$$ (23)

where ${\hat{\gamma }}_{\text{0}}$ denotes the variance of the loss differential series and ${\hat{\gamma }}_{k}$ denotes the sample autocovariance at lag k. The horizon parameter is set as h=1,2,3 for Step 1, Step 2, and Step 3, respectively, with autocovariances included up to lag h−1.

The null hypothesis of the DM test assumes that the two models have equal forecasting accuracy:


$$\begin{array}{c} H_{\text{0}}:E\left(d_{t}\right)=0 \end{array}$$ (24)

Since this study focuses on whether TSGAN achieves a lower forecasting loss than the baseline model, a one-sided test is adopted, with the alternative hypothesis defined as:


$$\begin{array}{c} H_{\text{1}}:E\left(d_{t}\right)<0 \end{array}$$ (25)

The DM test is conducted separately for each forecasting horizon, without pooling the error series across horizons. To address multiple comparisons, Holm’s step-down correction is applied separately within each dataset, treating all pairwise comparisons across baseline models, quarters, and forecasting horizons as one hypothesis family [38]. Let $p_{\left(1\right)}\leq ...\leq p_{\left(m\right)}$ denote the ordered, unadjusted one-sided p-values within a dataset, where m is the number of comparisons. The adjusted p-values are calculated as:


$$\begin{array}{c} p_{\left(i\right)}^{\text{Holm}}\text{=}min\left\{1,\underset{1\leq j\leq i}{max}\left[m-j+1)p_{\left(j\right)}\right]\right\},\;i=1,...,m \end{array}$$ (26)

The adjusted p-values are then mapped back to their corresponding comparisons. The reported DM statistics remain unchanged, while significance at the 1%, 5%, and 10% levels is determined using the Holm-adjusted p-values.

## 3 Empirical research

### 3.1 Data sources and sample construction

This paper conducts experiments using an open dataset published on the Open Energy Data Initiative (OEDI) platform. The data are sourced from the *Albuquerque _data* and the *Eugene_data* subsets available on its public webpage (https://data.openei.org/submissions/8414) [39]. These datasets are designed for the joint modeling of solar irradiance and sky images. They include ground-based meteorological observations, solar irradiance variables, and sky images corresponding to the observation times at the Albuquerque, New Mexico, and Eugene, Oregon sites, thereby providing both numerical state information and visual information on cloud conditions. This study uses the Albuquerque 2021 and Eugene 2024 datasets for model development and evaluation. Both datasets provide aligned solar irradiance observations, meteorological variables, and sky images and have been widely used in solar forecasting research. Moreover, Albuquerque and Eugene represent different geographical locations, while the selected data are from different years. Using data from different sites and years therefore provides a more comprehensive evaluation of the robustness and generalization capability of the proposed model under different irradiance distributions and cloud conditions. The data from each site are further divided into four subsets, Q1, Q2, Q3, and Q4, based on calendar quarters to evaluate forecasting performance under different seasonal conditions.

This study uses data with a 10-minute resolution to ensure temporal consistency across different modalities. Numerical variables and corresponding sky images are aligned according to the same time scale and used as inputs for the forecasting model. After this processing, each sample contains both a historical numerical sequence and a sky-image sequence with the same time index, thereby ensuring temporal consistency across modalities. For missing values and invalid samples, this study first excludes records that cannot be matched to valid image paths and interpolates isolated missing values in the numerical variables. Additionally, considering that the forecasting task has limited practical significance when nighttime irradiance is zero, this study retains only valid daytime samples for which ghi_0 > 0, preventing a large number of nighttime zero-value samples from weakening the model’s ability to learn cloud-induced irradiance fluctuations.

Since the nighttime period typically corresponds to a fixed state of zero irradiance, directly including it in model training and evaluation may cause the model to achieve a lower error by simply predicting zero values, thereby skewing the assessment of the model’s ability to accurately predict actual daytime irradiance variations. Furthermore, daytime filtering does not affect the practical application value of a continuous 24-hour forecasting system. In real-world operational scenarios, the objective of GHI forecasting during the nighttime period is typically to maintain a zero-irradiance state, rather than to predict complex weather-driven variations; therefore, evaluating nighttime zero-value samples alongside daytime dynamic irradiance samples may actually obscure the model’s ability to predict critical daytime variation processes. For continuous 24-hour operational systems, the transition between nighttime state predictions and daytime irradiance predictions can be achieved by combining day-night state identification or simple nighttime zero-value rule processing. Therefore, the daytime-only filtering method described in this paper is primarily used to improve the effectiveness of evaluating ultra-short-term daytime irradiance changes, without altering the actual deployment logic of the all-weather prediction system.

Regarding variable selection, this study uses global horizontal irradiance, ghi_0, as the forecasting target and employs its historical observations as key input information for the numerical modality. The numerical modality primarily includes variables related to irradiance, meteorological conditions, and local wind fields, which are used to characterize variations in solar irradiance and their external weather drivers. The image modality consists of sky images corresponding to the observation times, which provide visual information such as cloud cover, cloud type, solar occlusion, and cloud texture. These two modalities characterize irradiance variation processes from the perspectives of structured meteorological observations and unstructured sky images, respectively, laying the foundation for subsequent multimodal fusion forecasting. The specific variable names and definitions are presented in Table 1.

**Table 1:** Description of numerical meteorological and irradiance variables.

Variable	The full name of the variable
Ghi_0	Global Horizontal Irradiance at the current time step
Dni_0	Direct Normal Irradiance at the current time step
Dhi_0	Diffuse Horizontal Irradiance at the current time step
Bp_0	Barometric Pressure at the current time step
Wind_speed_0	Wind speed at the current time step
Wind_direction_0	Wind direction at the current time step
Temp_air_0	Air temperature at the current time step
Relative_humidity_0	Relative humidity at the current time step
Wind_direction_std	Standard deviation of wind direction at the current time step
Rain_mm_tot	Total accumulated rainfall at the current time step

During the sample construction phase, this paper uses a sliding-window approach to generate supervised learning samples. The aggregated data have a time interval of 10 minutes, and the model uses observations from the previous hour as input; thus, each sample contains numerical features from six consecutive historical time steps and the corresponding sky images. The prediction targets are global horizontal irradiance values for the next 10, 20, and 30 minutes, corresponding to forecasting steps h = 1, 2, and 3, respectively.

To avoid information leakage in time-series forecasting, all datasets in this paper are partitioned chronologically rather than randomly shuffled, thereby preserving the temporal structure of the samples. Within each quarterly dataset, the first 70% of the samples are used for model training, the subsequent 15% for validation and parameter selection, and the final 15% for testing and performance evaluation. To further prevent leakage caused by overlapping sliding windows, sliding-window samples are constructed separately within each subset after chronological partitioning, ensuring that no input window crosses the boundaries between the training, validation, and test sets.

To validate the effectiveness of the proposed multimodal gated fusion model, this paper establishes three comparison groups. Comparison Group (i) compares the proposed method with baseline models that use only the numerical modality, including traditional statistical models, machine learning models, and deep time-series models, to assess whether introducing sky images can further improve forecasting accuracy at different forecasting horizons. Comparison Group (ii) compares the proposed method with visual forecasting models that use only sky images to examine whether combining numerical sequence information with image information improves the ability to characterize absolute irradiance levels and capture cloud-induced fluctuations. Comparison Group (iii) compares the proposed method with multimodal fusion strategies, such as direct concatenation, standard gated fusion, and cross-attention, to verify the effectiveness of TSGAN in adaptive modality selection and forecasting performance enhancement.

For numerical inputs, missing value handling and normalization are performed before model training. Records that cannot be matched with valid image paths are removed, and isolated missing values in numerical variables are interpolated using linear interpolation. In this study, an isolated missing value refers to an individual missing observation or a short missing interval surrounded by valid observations before and after the missing timestamp. Samples containing continuous missing segments that prevent the construction of complete temporal input windows are excluded. Subsequently, numerical variables are standardized using the Z-score normalization method:


$$\begin{array}{c} x^{\prime }=\frac{x-\mu }{\sigma } \end{array}$$ (27)

where x denotes the original numerical variable, and μ and σ represent the mean and standard deviation, respectively. Both the numerical input variables and the target GHI values are standardized using Z-score normalization. The corresponding means and standard deviations are estimated using the training set only and subsequently applied to the validation and test sets to avoid information leakage. The predicted GHI values are inverse-transformed to the original scale before the evaluation metrics are calculated.

For image inputs, all sky images are first resized to 128×128 pixels and normalized by scaling RGB pixel values to the range of [0,1]. No additional data augmentation strategies, such as random rotation, cropping, or color transformation, are applied to preserve the physical characteristics of cloud structures and solar occlusion states.

The model training process is configured as follows. The proposed model was trained using the AdamW optimizer by minimizing the MSE loss with an initial learning rate of 1 × 10^−4^, a weight decay of 1 × 10^−4^, a batch size of 16, and a maximum of 30 epochs. The numerical Transformer encoder consisted of two layers, while the image branch employed a single-layer temporal Transformer. Both branches used four attention heads with an embedding dimension of 128, and the feed-forward dimension of each Transformer encoder was set to 512. The dropout rate was set to 0.05. Early stopping with a patience of 10 epochs was applied according to the validation RMSE. To ensure reproducibility, the random seed was fixed at 2026 to improve the reproducibility of the experiments.

For completeness, the trainable parameters of each component are summarized in Table 2 to improve the reproducibility of the proposed framework. The parameter counts are calculated by summing all learnable weights and biases of the corresponding layers. The table provides the detailed parameter distribution of each module, including the numerical Transformer encoder, image encoder, state-aware fusion module, prediction decoder, and the complete TSGAN model.

**Table 2:** Trainable parameter statistics of TSGAN.

Module	Main configuration	Trainable parameter
Numerical Transformer encoder	$d_{model}=128$, 4 attention heads, 2 Transformer encoder layers	397,952
CNN backbone	4 convolutional blocks, channels: 32-64-128-256, 3×3 kernels	422,272
Image temporal Transformer	$d_{model}=128$, 4 attention heads, 1 Transformer encoder layer	198,528
State-aware fusion module	Gated attention fusion structure	197,890
Prediction decoder	Fully connected layers: 128-128-64-1	24,833
**Total** TSGAN	—	**1,241,475**

### 3.2 Comparison with the baseline model

To evaluate the effectiveness of the proposed method compared with conventional numerical forecasting frameworks, this paper selects XGBoost, RF, MLP, GRU, LSTM, Transformer, and PatchTST as baseline models [40–42]. Among these, RF, XGBoost, and MLP represent traditional machine learning and ensemble learning methods; GRU and LSTM focus on extracting recurrent dependencies within sequences; and Transformer and PatchTST further characterize time series from the perspectives of global dependencies and patch-based modeling, respectively. All models employ the same data partitioning, input window, and forecasting settings; the relevant results are presented in Tables 3–4, and Fig 2.

**Table 3:** Performance comparison between the proposed model and single numerical modality models for solar irradiance forecasting on the Albuquerque dataset.

STEP	Model	Q1				Q2				Q3				Q4			
		MAE	SMAPE	NRMSE	R^2^	MAE	SMAPE	NRMSE	R^2^	MAE	SMAPE	NRMSE	R^2^	MAE	SMAPE	NRMSE	R^2^
Step-1	RF	52.9897	12.3797	15.3156	0.9256	56.3470	16.8027	19.6662	0.9216	47.7288	16.7416	17.5993	0.9212	35.4693	19.8229	22.5299	0.9017
	XGBoost	58.7660	14.0933	16.4614	0.9140	63.4248	19.6014	20.5291	0.9146	51.5024	19.1569	17.5571	0.9216	38.9485	23.8233	23.1045	0.8966
	MLP	52.5641	17.5571	15.2730	0.9260	56.1962	22.6262	19.2813	0.9246	68.2113	26.8714	20.5997	0.8921	39.4765	23.9402	23.3823	0.8941
	GRU	40.0042	13.5983	13.2609	0.9442	53.9849	21.2596	18.9924	0.9269	61.1701	25.7836	19.3013	0.9052	36.1166	22.9691	21.6926	0.9089
	LSTM	46.8536	15.4118	13.6882	0.9405	53.5547	19.9720	18.8822	0.9277	54.3577	20.0967	18.7509	0.9106	38.8054	26.6381	22.4317	0.9025
	Transformer	47.2497	14.6467	13.9456	0.9383	52.4942	18.2871	18.7854	0.9285	58.2745	24.1689	18.6239	0.9118	41.7129	27.0208	23.6987	0.8912
	PatchTST	46.2377	13.8455	14.3517	0.9346	53.1004	21.6201	18.9871	0.9269	50.9296	20.3758	17.7784	0.9196	36.2664	24.5423	22.3933	0.9029
	**TSGAN**	**28.4211**	**10.7657**	**9.5866**	**0.9708**	**36.6041**	**20.1119**	**12.6762**	**0.9674**	**36.1998**	**14.6895**	**12.3918**	**0.9609**	**24.5830**	**17.4334**	**15.2121**	**0.9552**
Step-2	RF	70.2633	17.6600	19.1439	0.8796	87.3365	26.7932	26.4605	0.8552	74.8056	25.8801	24.9931	0.8371	56.4977	32.7474	31.8323	0.7988
	XGBoost	77.2864	19.4473	20.5321	0.8615	88.1219	28.0325	26.2850	0.8571	79.7950	28.2082	25.0105	0.8369	57.5884	34.4438	32.0023	0.7967
	MLP	74.9282	20.9336	19.3706	0.8768	87.5695	31.2376	25.2535	0.8681	90.8654	32.1935	28.3509	0.7904	57.1429	35.2786	31.5829	0.8020
	GRU	59.0432	19.2945	16.9440	0.9057	80.8119	31.3712	24.1437	0.8794	75.5947	29.0572	24.7177	0.8407	50.0470	32.7457	28.6730	0.8368
	LSTM	70.0936	20.0605	18.3628	0.8893	83.3903	29.1944	24.8387	0.8724	87.0310	32.1071	26.6532	0.8147	57.9969	34.8493	30.7947	0.8117
	Transformer	70.2039	19.7134	18.9135	0.8825	74.6050	27.0350	24.3105	0.8778	87.6198	31.7198	25.8468	0.8258	53.7978	33.9544	32.5558	0.7896
	PatchTST	61.6895	20.1016	17.8046	0.8959	80.5617	29.7874	25.2363	0.8683	75.8673	28.7317	23.7668	0.8527	63.0903	38.0866	34.8760	0.7585
	**TSGAN**	**43.9107**	**15.0788**	**14.2682**	**0.9331**	**59.2346**	**22.4698**	**20.5965**	**0.9123**	**49.3443**	**22.3576**	**15.4098**	**0.9381**	**38.5968**	**24.3409**	**24.6437**	**0.8794**
Step-3	RF	88.8093	21.8896	22.5330	0.8283	114.8431	33.7175	31.9194	0.7854	92.7084	30.7261	30.0340	0.7594	69.5711	39.0298	37.1546	0.7200
	XGBoost	95.1660	23.1660	23.8095	0.8083	115.9998	35.5146	31.7069	0.7883	96.1837	32.8281	29.8207	0.7628	74.8514	42.0306	38.9493	0.6923
	MLP	89.9705	26.7531	22.3870	0.8305	117.1338	39.0895	33.1054	0.7692	106.2680	37.0357	30.9844	0.7439	66.7619	40.8752	35.7077	0.7414
	GRU	73.5781	21.8581	20.6928	0.8552	104.8188	38.3769	29.1331	0.8213	96.5297	34.3541	29.8170	0.7628	66.5768	40.3966	33.3098	0.7750
	LSTM	80.7717	23.0682	21.1303	0.8490	114.9874	38.3904	30.5616	0.8033	104.5444	35.7512	30.9886	0.7438	68.1873	41.8047	35.9558	0.7378
	Transformer	81.1241	22.0003	21.2581	0.8472	96.5460	34.4869	28.3348	0.8309	95.2539	33.3290	29.5062	0.7678	66.8385	38.5422	35.3214	0.7470
	PatchTST	78.5047	23.6689	21.7759	0.8396	84.8937	31.4819	27.9287	0.8357	98.9263	34.3361	29.5281	0.7674	70.7175	38.8915	39.6316	0.6814
	**TSGAN**	**59.0974**	**20.7958**	**16.8688**	**0.9038**	**79.5132**	**29.5678**	**24.2747**	**0.8759**	**64.6891**	**25.3517**	**21.3197**	**0.8788**	**47.5525**	**31.9790**	**26.5137**	**0.8574**

**Table 4:** Performance comparison between the proposed model and single numerical modality models for solar irradiance forecasting on the Eugene dataset.

STEP	Model	Q1				Q2				Q3				Q4			
		MAE	SMAPE	NRMSE	R^2^	MAE	SMAPE	NRMSE	R^2^	MAE	SMAPE	NRMSE	R^2^	MAE	SMAPE	NRMSE	R^2^
Step-1	RF	91.5649	32.0523	46.6924	0.6754	46.6672	**12.8464**	20.9141	0.8840	53.6938	**16.5003**	26.9403	0.8324	27.5413	**30.2781**	52.3660	0.6834
	XGBoost	93.0310	32.1151	47.0128	0.6709	52.8947	13.9622	21.1008	0.8819	56.2825	17.2865	26.8449	0.8336	27.2180	29.9098	51.8539	0.6895
	MLP	83.0193	31.8065	45.3472	0.6939	61.6775	18.1074	22.1231	0.8702	70.3276	25.1431	28.7531	0.8091	29.2470	37.4332	52.9801	0.6759
	GRU	75.9515	31.1682	40.0949	0.7607	56.0263	15.7826	21.0838	0.8821	66.8694	23.6246	28.3221	0.8148	28.8074	35.5461	53.4714	0.6699
	LSTM	79.0648	31.8322	40.7666	0.7526	61.7765	19.2554	21.8292	0.8736	61.4021	24.1678	27.0856	0.8306	27.9458	32.4756	52.5037	0.6817
	Transformer	81.9484	30.6875	40.4235	0.7567	62.9632	17.4790	21.2220	0.8806	67.3166	26.8938	27.0735	0.8307	28.9441	36.7776	52.0307	0.6874
	PatchTST	78.0173	32.1080	39.8323	0.7638	57.2577	16.4582	20.8871	0.8843	71.0143	25.7183	28.0164	0.8187	28.7924	33.5247	54.6573	0.6551
	**TSGAN**	**64.3873**	**28.5700**	**35.3055**	**0.8144**	**41.2731**	18.5214	**16.0634**	**0.9316**	**50.6590**	24.0737	**21.8624**	**0.8896**	**22.5640**	30.5794	**40.9926**	**0.8060**
Step-2	RF	111.6720	40.2243	54.6300	0.5490	59.8624	**17.6994**	23.2291	0.8530	69.3027	**22.3790**	31.7591	0.7622	37.2296	41.5326	63.8461	0.5240
	XGBoost	116.7450	41.1615	56.8207	0.5121	65.3174	18.1941	23.2992	0.8521	70.4311	22.9162	31.2986	0.7690	36.9604	**41.3547**	64.1104	0.5201
	MLP	112.2284	39.6739	62.6537	0.4068	80.5152	24.1910	24.7183	0.8335	85.6205	28.1889	34.7628	0.7150	37.6100	45.0591	64.8045	0.5096
	GRU	102.1808	44.3247	49.8581	0.6243	70.9001	20.5927	23.3962	0.8509	82.1130	27.3658	34.0473	0.7266	36.8012	43.8202	64.3909	0.5158
	LSTM	95.4522	38.6546	45.3750	0.6889	73.9666	21.2670	23.9876	0.8432	86.6391	28.2211	35.5066	0.7027	37.0052	45.1026	63.6476	0.5270
	Transformer	98.8201	39.3051	47.3084	0.6618	72.6844	20.8415	23.7298	0.8466	84.5439	29.4945	33.3050	0.7384	37.3750	42.0760	64.8039	0.5096
	PatchTST	98.8328	41.6149	47.2997	0.6619	91.4952	25.0831	25.6334	0.8210	88.2166	30.0523	34.5981	0.7177	37.7976	45.7685	64.6779	0.5115
	**TSGAN**	**84.8537**	**33.2236**	**41.6684**	**0.7376**	**55.6634**	18.7006	**19.7843**	**0.8934**	**68.8343**	26.3776	**28.4239**	**0.8095**	**29.8456**	43.7136	**51.9286**	**0.6851**
Step-3	RF	130.1649	47.1135	61.8421	0.4123	66.7245	19.9461	25.1971	0.8227	82.4181	27.0440	35.1783	0.7027	44.5197	**49.0238**	72.1544	0.3873
	XGBoost	128.6547	46.5571	60.6772	0.4343	74.8516	20.7547	25.4533	0.8191	80.4067	**26.0858**	33.4265	0.7316	44.3404	49.1624	72.0158	0.3896
	MLP	120.8253	47.6934	57.1395	0.4983	83.8961	23.5876	26.6870	0.8012	100.4633	31.3638	39.7649	0.6201	46.2770	54.4899	73.9629	0.3562
	GRU	113.2696	47.6588	54.3116	0.5468	76.5059	22.2591	25.4205	0.8196	95.8166	29.6406	37.8152	0.6565	43.9513	52.7261	72.7598	0.3769
	LSTM	106.7121	43.1989	48.7989	0.6341	83.3164	24.6935	26.4563	0.8046	95.7937	30.9822	38.2413	0.6487	43.9499	52.8957	71.4229	0.3996
	Transformer	107.8256	43.1108	48.7590	0.6347	91.0009	23.5284	26.7791	0.7998	104.2239	37.5837	37.0545	0.6702	45.2352	51.4021	73.7535	0.3598
	PatchTST	103.6316	42.1391	48.3667	0.6405	82.7222	22.5471	25.4595	0.8190	97.2756	33.0240	37.4610	0.6629	45.3139	55.3018	72.1260	0.3877
	**TSGAN**	**91.0199**	**34.1640**	**46.4626**	**0.6683**	**62.7747**	**19.7667**	**20.9817**	**0.8771**	**74.2571**	28.0460	**31.5499**	**0.7609**	**40.6783**	54.2884	**62.8839**	**0.5346**

**Fig 2:**
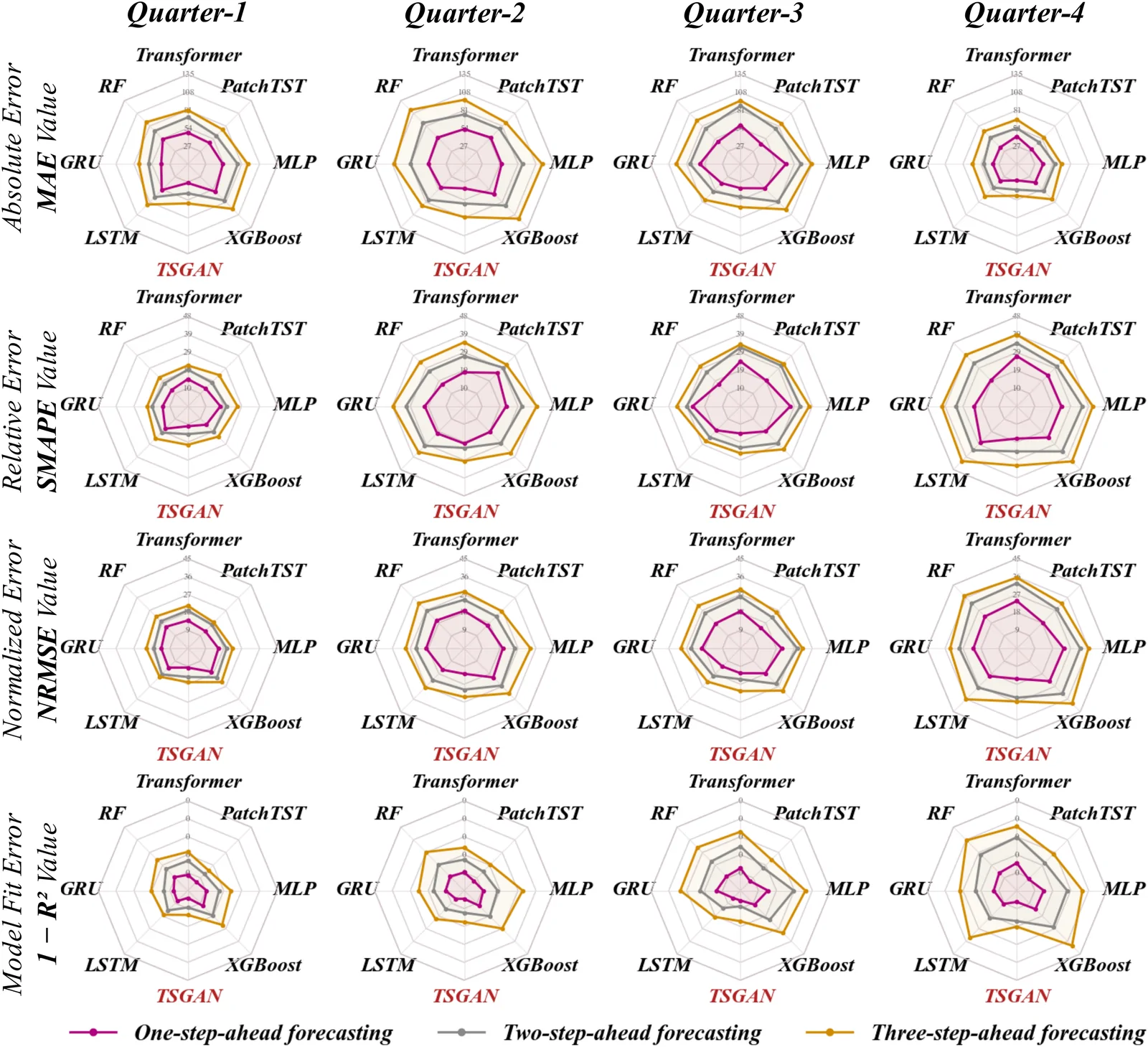
Performance comparison of numerical forecasting models under different forecasting horizons.

As shown by the performance distributions of the various models across different quarters and forecasting horizons in the figure, all types of numerical models are capable of learning certain variation patterns from historical irradiance and meteorological variables; however, their forecasting capabilities vary significantly across seasons and as the forecasting horizon increases. Across both the Albuquerque 2021 and Eugene 2024 datasets, the proposed method demonstrates superior overall forecasting performance. Specifically, for the Albuquerque 2021 dataset, TSGAN achieves average MAE, NRMSE, SMAPE, and $R^{\text{2}}$ values of 47.312, 17.813, 21.245, and 0.9194, respectively, while the corresponding values for the Eugene 2024 dataset are 57.234, 34.826, 30.002, and 0.7840, respectively. Although the two datasets exhibit differences in irradiance distributions and temporal variation characteristics, the proposed model still achieves the best or competitive performance in the vast majority of quarter–horizon evaluation settings. In particular, the Eugene dataset contains more pronounced short-term irradiance fluctuations, yet TSGAN is still able to maintain a clear overall advantage over the baseline models. This indicates that the proposed multimodal fusion framework can effectively adapt to changes in environmental and geographical conditions and retain its forecasting capability under more rapidly varying irradiance conditions. Further examination of performance variations across different forecasting horizons reveals that the proposed method demonstrates a more pronounced advantage in one-step-ahead forecasts. This is because irradiance changes over short timescales are often directly influenced by local cloud movement and shading variations, whereas visual information provides prior knowledge of the spatial state in the immediate vicinity, enabling the model to capture potential irradiance trends in advance. Compared with models that rely solely on historical numerical responses, the proposed method can utilize image information to promptly correct for sudden irradiance fluctuations, thereby achieving higher accuracy in short-term forecasting.

As the forecasting horizon extends from Step 1 to Step 3, performance generally declines. This is because irradiance variations are influenced not only by historical trends but also by short-term dynamic factors such as cloud movement and changes in shaded areas. For models that rely solely on numerical information, forecasts are primarily based on observed irradiance responses. When rapid cloud changes occur in the future, these models struggle to obtain timely information about changes in the external environment, making them prone to forecasting lags and cumulative errors. In contrast, although the performance of the proposed method also decreases as the forecasting horizon increases, it maintains an overall advantage over the numerical baseline models. For the Albuquerque 2021 dataset, the average $R^{\text{2}}$ decreases from 0.9636 at Step 1 to 0.8790 at Step 3, while for the Eugene 2024 dataset, it decreases from 0.8604 to 0.7102. Despite the stronger performance degradation observed on the Eugene dataset, which contains more pronounced short-term irradiance fluctuations, the proposed method still maintains competitive forecasting performance as the forecasting horizon increases. This indicates that multimodal information fusion can effectively enhance the model’s ability to anticipate future irradiance trends. Specifically, the numerical modality provides historical patterns of irradiance variation and meteorological constraints, while the image modality extracts early-warning information from cloud structures and potential occlusion trends. The two modalities complement each other, enabling the model not only to learn “changes that have already occurred” but also to capture “changes that are about to occur,” thereby reducing the uncertainty associated with extrapolation based solely on historical time series.

### 3.3 Comparison with single-modality image models

Furthermore, this paper compares the proposed method with single-image-modality models, including CNN, ResNet, and CNN-LSTM [43]. These models directly extract cloud-texture and temporal-evolution information from continuous sky-image sequences, providing visual cues regarding rapid changes in irradiance. All models use the same image sequences and data partitions; the relevant results are presented in Tables 5–6, and Fig 3.

**Table 5:** Comparison of the proposed model with image-based methods on the Albuquerque dataset.

STEP	Model	Q1				Q2				Q3				Q4			
		MAE	SMAPE	NRMSE	R^2^	MAE	SMAPE	NRMSE	R^2^	MAE	SMAPE	NRMSE	R^2^	MAE	SMAPE	NRMSE	R^2^
**Step-1**	CNN	60.8863	19.4475	14.9276	0.9293	46.4366	20.0451	15.0090	0.9543	49.2031	18.6164	15.0043	0.9427	39.3357	31.4015	20.9847	0.9147
	ResNet-LSTM	67.8866	19.7962	17.0178	0.9081	52.6929	20.2368	16.5982	0.9441	50.6120	21.8449	14.6862	0.9451	79.4117	43.8879	37.2124	0.7318
	CNN-LSTM	55.2556	17.1375	13.6226	0.9411	48.8529	20.3098	16.4731	0.9450	40.4415	18.3717	12.7693	0.9585	35.5252	27.4414	19.5867	0.9257
	ResNet-GRU	84.2449	23.0642	18.8898	0.8868	47.5070	19.2686	15.3606	0.9522	50.7806	21.0146	14.8617	0.9438	62.9657	47.7757	33.1964	0.7865
	CNN-GRU	60.1757	17.6644	14.3149	0.9350	49.7185	20.6398	15.1326	0.9536	45.5170	18.2212	14.6199	0.9456	40.0758	28.4571	24.7963	0.8809
	**TSGAN**	**28.4211**	**10.7657**	**9.5866**	**0.9708**	**36.6041**	**20.1119**	**12.6762**	**0.9674**	**36.1998**	**14.6895**	**12.3918**	**0.9609**	**24.5830**	**17.4334**	**15.2121**	**0.9552**
**Step-2**	CNN	75.3770	20.9309	18.3015	0.8900	71.1133	29.1468	22.1796	0.8982	60.5951	24.4050	20.9891	0.8851	51.6569	35.4326	28.4207	0.8396
	ResNet-LSTM	110.5658	29.9702	25.9004	0.7797	74.5637	27.0218	23.4731	0.8860	74.1428	28.7704	22.7781	0.8647	50.6950	36.7209	28.5991	0.8376
	CNN-LSTM	84.9361	21.5693	19.5844	0.8740	75.9351	30.0893	22.3264	0.8969	63.3491	23.9593	21.0501	0.8844	50.0196	33.5476	27.5993	0.8488
	ResNet-GRU	114.3686	34.1274	30.5537	0.6934	80.7047	28.2466	24.4027	0.8768	71.7284	25.6310	21.5609	0.8788	56.1173	38.5184	31.1703	0.8071
	CNN-GRU	102.9355	28.5014	24.3959	0.8045	73.2372	28.5925	23.1134	0.8895	57.9530	23.5628	19.3614	0.9022	50.1919	32.3101	28.9391	0.8337
	**TSGAN**	**43.9107**	**15.0788**	**14.2682**	**0.9331**	**59.2346**	**22.4698**	**20.5965**	**0.9123**	**49.3443**	**22.3576**	**15.4098**	**0.9381**	**38.5968**	**24.3409**	**24.6437**	**0.8794**
**Step-3**	CNN	83.5889	23.2879	20.1045	0.8633	86.1066	33.8267	25.8874	0.8589	77.5286	30.0210	23.3805	0.8542	67.7925	42.3373	33.8331	0.7678
	ResNet-LSTM	90.1304	27.1278	20.7855	0.8539	103.8583	37.2314	30.2723	0.8070	82.9596	30.0554	23.9282	0.8473	67.9225	41.4289	34.9518	0.7522
	CNN-LSTM	85.9227	24.7037	19.9498	0.8654	105.5398	36.7112	29.5436	0.8162	76.0604	26.6169	23.4054	0.8539	71.1775	42.0150	36.2762	0.7331
	ResNet-GRU	109.9737	29.0672	25.5261	0.7796	86.6949	31.7819	26.1368	0.8561	89.3834	32.2895	26.5124	0.8125	67.0376	39.0241	33.6354	0.7705
	CNN-GRU	89.6667	25.8159	21.3057	0.8465	88.9447	34.8601	27.5067	0.8407	73.2801	26.1237	23.7445	0.8496	76.8135	44.3453	37.3280	0.7174
	TSGAN	**59.0974**	**20.7958**	**16.8688**	**0.9038**	**79.5132**	**29.5678**	**24.2747**	**0.8759**	**64.6891**	**25.3517**	**21.3197**	**0.8788**	**47.5525**	**31.9790**	**26.5137**	**0.8574**

**Table 6:** Comparison of the proposed model with image-based methods on the Eugene dataset.

STEP	Model	Q1				Q2				Q3				Q4			
		MAE	SMAPE	NRMSE	R^2^	MAE	SMAPE	NRMSE	R^2^	MAE	SMAPE	NRMSE	R^2^	MAE	SMAPE	NRMSE	R^2^
**Step-1**	CNN	88.9717	36.4207	42.5924	0.7299	56.4446	20.1359	19.3449	0.9008	87.7056	30.0900	28.5485	0.8118	37.5584	49.7233	62.4625	0.5495
	ResNet-LSTM	82.0234	33.2241	39.0360	0.7731	63.0871	22.7257	20.1357	0.8925	86.8608	33.0325	29.5433	0.7984	40.0642	51.5038	62.8912	0.5433
	CNN-LSTM	76.1702	34.2514	38.5410	0.7789	67.3936	21.4696	22.2138	0.8691	93.3365	32.3023	32.9860	0.7487	38.2669	49.0829	57.5552	0.6175
	ResNet-GRU	85.4329	33.0096	41.0039	0.7497	62.0372	20.0950	20.1853	0.8920	88.0357	31.2435	29.8151	0.7947	40.8476	51.4681	65.7496	0.5008
	CNN-GRU	90.1714	36.4410	41.3178	0.7458	65.2709	22.9560	20.0486	0.8934	90.8377	33.5361	30.4600	0.7857	36.0161	47.4320	56.2051	0.6352
	**TSGAN**	**64.3873**	**28.5700**	**35.3055**	**0.8144**	**41.2731**	**18.5214**	**16.0634**	**0.9316**	**50.6590**	**24.0737**	**21.8624**	**0.8896**	**22.5640**	**30.5794**	**40.9926**	**0.8060**
**Step-2**	CNN	92.7062	38.7026	44.4495	0.7014	76.7400	26.2417	22.0730	0.8673	82.4079	31.9971	29.6979	0.7920	44.2459	56.4118	64.6222	0.5124
	ResNet-LSTM	99.8348	38.7534	47.2561	0.6625	67.2263	21.8864	23.0224	0.8556	89.8333	33.0915	32.4356	0.7519	48.3595	59.4841	67.1071	0.4741
	CNN-LSTM	95.1615	40.2967	46.5535	0.6725	80.7661	23.0274	22.1702	0.8661	84.6307	32.8825	30.7613	0.7769	44.8485	56.0737	66.6971	0.4805
	ResNet-GRU	122.6292	50.1865	50.0135	0.6220	65.8241	22.7468	21.3942	0.8753	92.7439	36.6013	33.0488	0.7424	49.6407	59.2127	72.2341	0.3907
	CNN-GRU	112.3109	49.4311	50.6201	0.6128	62.3763	22.0584	21.7344	0.8713	91.6596	33.2599	32.0262	0.7581	46.8577	53.0913	68.2764	0.4557
	**TSGAN**	**84.8537**	**33.2236**	**41.6684**	**0.7376**	**55.6634**	**18.7006**	**19.7843**	**0.8934**	**68.8343**	**26.3776**	**28.4239**	**0.8095**	**29.8456**	**43.7136**	**51.9286**	**0.6851**
**Step-3**	CNN	105.3835	42.5864	47.3168	0.6560	68.7224	22.5453	22.4120	0.8598	100.0853	36.3253	34.8654	0.7080	55.0516	63.6812	78.1153	0.2818
	ResNet-LSTM	110.1569	43.0885	48.3319	0.6411	72.4312	23.8321	22.7973	0.8549	92.6411	33.5592	33.3697	0.7325	66.9906	73.1506	82.4074	0.2008
	CNN-LSTM	103.5528	42.1837	46.9261	0.6616	83.8801	26.6774	24.6404	0.8305	92.0438	35.1484	34.0898	0.7208	52.5380	63.1076	70.2683	0.4189
	ResNet-GRU	110.1599	46.5297	49.9260	0.6170	78.5194	24.2576	23.6138	0.8443	111.8378	41.3181	35.5303	0.6967	54.4450	63.3898	75.4034	0.3308
	CNN-GRU	128.2020	48.7073	53.1720	0.5656	75.2634	26.2392	23.6348	0.8440	100.1368	36.9024	34.8367	0.7085	49.8148	59.8058	69.7125	0.4280
	TSGAN	**91.0199**	**34.1640**	**46.4626**	**0.6683**	**62.7747**	**19.7667**	**20.9817**	**0.8771**	**74.2571**	**28.0460**	**31.5499**	**0.7609**	**40.6783**	**54.2884**	**62.8839**	**0.5346**

**Fig 3:**
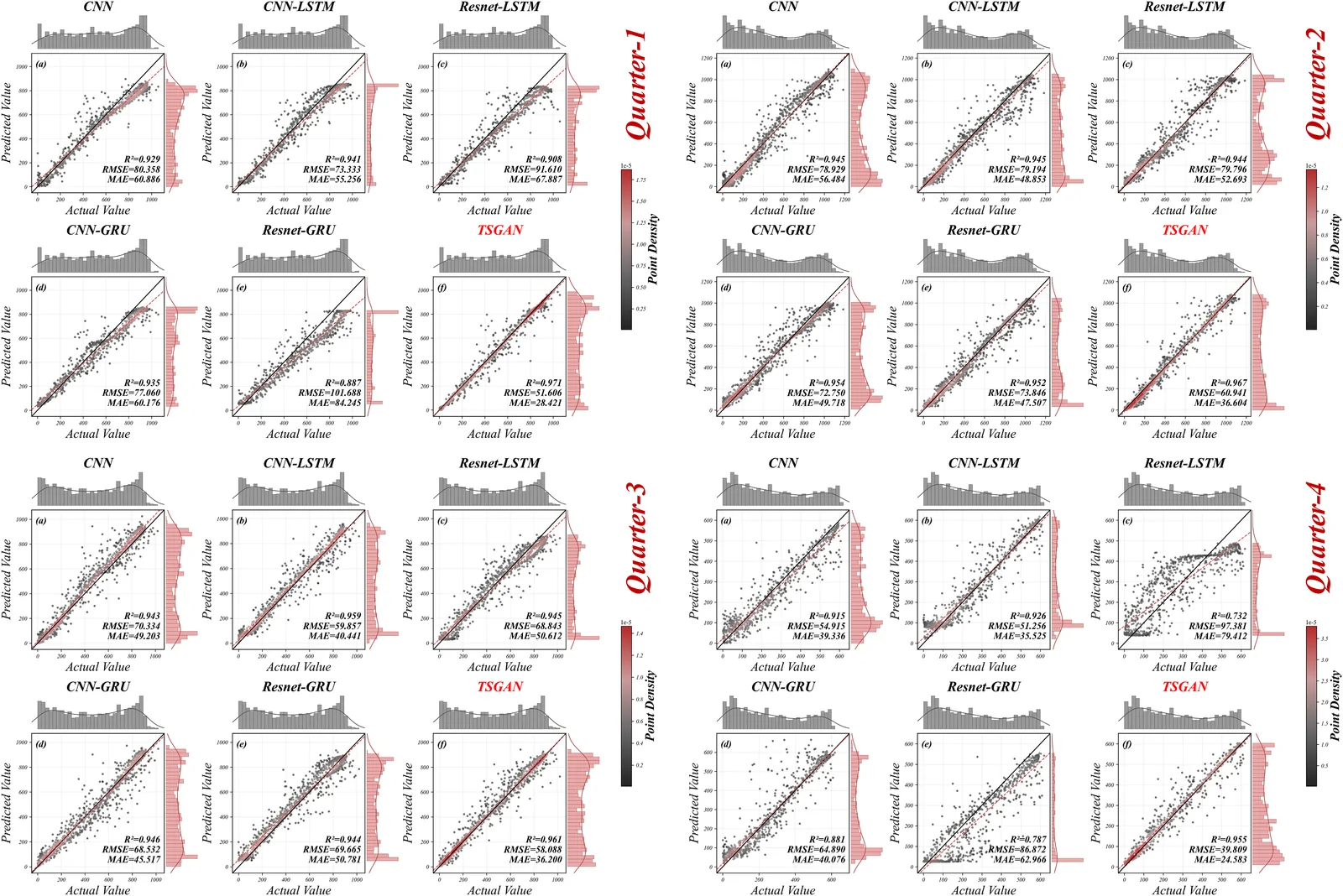
Performance comparison of image-based forecasting models. Scatter plots comparing predicted and observed irradiance values for different image-based forecasting models across four quarters. The black solid line denotes the ideal line y = x, the red dashed line denotes the linear regression line, the top and right marginal histograms show the distributions of observed and predicted values, respectively, and the color indicates point density.

The figure compares the forecasting results of the proposed method and the single-image-modality models across different quarters. The scatter plots illustrate the agreement between the predicted and actual irradiance values, while the black dashed line represents perfect agreement. The closer the data points are to this line, the more closely the model’s predictions match the actual values. The marginal histograms at the top, bottom, and right sides of the figure show the distributions of actual and predicted irradiance values across different ranges. These histograms can be used to assess whether the model captures the variation range and probability distribution of actual irradiance.

As shown in the figure, although single-image models such as CNN, ResNet, and CNN-LSTM can capture certain trends in irradiance variation by utilizing cloud structures and occlusion changes in sky images, their prediction points still exhibit some dispersion, with some deviating significantly from the ideal diagonal line. At the same time, discrepancies exist between the distributions of the predicted and actual values, indicating that when relying solely on visual information, the models’ ability to characterize the overall range of irradiance variation remains limited. This is primarily because image information reflects the current state of the sky, whereas irradiance is determined not only by cloud morphology but also by factors such as the sun’s position and historical irradiance conditions. Identical or similar visual features may correspond to different irradiance responses, making single-image models prone to forecasting biases in complex environments. In contrast, the prediction points of the proposed method are generally more concentrated around the ideal diagonal line and exhibit greater consistency across different quarters. Furthermore, the distribution of its predicted values more closely resembles that of the actual values, indicating that the model not only reduces forecasting errors but also more accurately preserves the overall distributional characteristics of irradiance variation. This suggests that incorporating additional state information can mitigate the uncertainty introduced by a single visual representation, resulting in more stable forecasts across different irradiance levels.

The results in the table show that, compared with the image-based baseline models, the proposed method achieves clear performance improvements on both the Albuquerque 2021 and Eugene 2024 datasets. Specifically, the proposed method reduces MAE and NRMSE by 32.38% and 23.32% on the Albuquerque dataset, and by 25.98% and 17.02% on the Eugene dataset, respectively. These results further demonstrate that multimodal fusion can effectively address the limitations of single-image models in characterizing irradiance states, enabling the model to capture trends more accurately and produce more reasonable forecast distributions. From this perspective, the improvement achieved by the proposed method does not simply stem from an increase in the amount of input information but rather from the complementary constraints formed between different types of information. While single-image models primarily rely on visual patterns to determine changes in irradiance, the proposed method integrates additional information related to irradiance conditions, enabling the model to obtain a more comprehensive understanding of variation patterns under complex weather conditions. Consequently, multimodal fusion enhances the model’s adaptability to abnormal fluctuations and different seasonal conditions, thereby improving the reliability of the forecasting results.

### 3.4 Comparison with other multimodal fusion methods

To further analyze the impact of the fusion strategy itself, this paper compares the proposed method with four representative multimodal fusion approaches, including the concatenation fusion method (CFN), Cross-Attention fusion method (CAFN), selective gated fusion method (SGFN), and Transformer-based Cross-Attention fusion method (TCAFN) [36]. Since the only differences among these methods lie in the modalities’ interaction and joint representation, this comparison allows us to evaluate the effectiveness of the fusion mechanisms while essentially eliminating differences in the input information. The relevant results are shown in Tables 7 and 8.

**Table 7:** Comparison of the proposed model with other fusion methods on the Albuquerque dataset.

STEP	Model	Q1				Q2				Q3				Q4			
		MAE	SMAPE	NRMSE	R^2^	MAE	SMAPE	NRMSE	R^2^	MAE	SMAPE	NRMSE	R^2^	MAE	SMAPE	NRMSE	R^2^
**Step-1**	CFN	33.8629	11.7939	11.2062	0.9602	43.5615	20.4751	13.9736	0.9604	40.3441	18.3517	12.7362	0.9587	25.4541	16.7763	16.3730	0.9481
	CAFN	38.5501	12.2260	11.0842	0.9610	49.3951	22.0236	15.1711	0.9533	42.4327	17.5376	13.5038	0.9536	32.0827	21.2089	20.3811	0.9195
	SGFN	32.3662	12.5895	11.1414	0.9606	39.5424	15.6119	14.1757	0.9593	40.4458	18.2122	13.2014	0.9557	28.5172	19.3743	17.8619	0.9382
	TCAFN	45.1537	13.5854	12.2421	0.9524	56.3061	25.4211	16.3367	0.9459	43.3537	19.5663	14.4369	0.9470	31.4102	23.6310	18.2674	0.9354
	**TSGAN**	**28.4211**	**10.7657**	**9.5866**	**0.9708**	**36.6041**	**20.1119**	**12.6762**	**0.9674**	**36.1998**	**14.6895**	**12.3918**	**0.9609**	**24.5830**	**17.4334**	**15.2121**	**0.9552**
**Step-2**	CFN	60.7973	18.0223	16.0775	0.9151	74.3315	27.6418	22.3132	0.8970	69.1981	26.5466	21.5787	0.8786	44.1622	29.3326	26.6717	0.8588
	CAFN	53.8047	17.2609	15.1621	0.9245	73.0267	28.9266	21.4064	0.9052	67.3755	27.6483	21.1848	0.8830	43.8535	28.5826	25.8506	0.8673
	SGFN	53.8722	20.7055	15.0869	0.9252	64.5147	23.6376	21.1750	0.9073	81.1567	31.3069	24.0044	0.8497	43.2551	27.6386	26.7824	0.8576
	TCAFN	60.4293	18.1699	16.7945	0.9074	74.8670	29.4463	22.6267	0.8941	74.9146	30.9109	22.0610	0.8731	46.6308	30.4362	27.9863	0.8445
	**TSGAN**	**43.9107**	**15.0788**	**14.2682**	**0.9331**	**59.2346**	**22.4698**	**20.5965**	**0.9123**	**49.3443**	**22.3576**	**15.4098**	**0.9381**	**38.5968**	**24.3409**	**24.6437**	**0.8794**
**Step-3**	CFN	71.9797	20.1198	18.6624	0.8822	86.3303	31.9057	25.1329	0.8670	96.4355	33.7121	27.5757	0.7972	53.0098	37.6579	31.2857	0.8015
	CAFN	89.4325	22.3617	20.9417	0.8517	81.4223	27.3142	25.0953	0.8674	86.5868	32.5640	25.4438	0.8273	62.3322	39.2589	33.5386	0.7719
	SGFN	66.3045	21.0048	17.5596	0.8957	**73.3572**	**27.4594**	**22.5308**	**0.8931**	90.1218	33.1891	25.7172	0.8236	58.3039	35.1412	32.8347	0.7813
	TCAFN	73.4771	23.7718	18.3604	0.8860	88.3074	31.9208	26.3327	0.8540	76.7755	27.8649	24.0623	0.8456	57.1233	34.4008	31.6957	0.7962
	**TSGAN**	**59.0974**	**20.7958**	**16.8688**	**0.9038**	79.5132	29.5678	24.2747	0.8759	**64.6891**	**25.3517**	**21.3197**	**0.8788**	**47.5525**	**31.9790**	**26.5137**	**0.8574**

**Note:** CFN represents the concatenation fusion method; CAFN represents the Cross-Attention fusion method; SGFN represents the selective gated fusion method; and TCAFN represents the Transformer-based Cross-Attention fusion method.

**Table 8:** Comparison of the proposed model with other fusion methods on the Eugene dataset.

STEP	Model	Q1				Q2				Q3				Q4			
		MAE	SMAPE	NRMSE	R^2^	MAE	SMAPE	NRMSE	R^2^	MAE	SMAPE	NRMSE	R^2^	MAE	SMAPE	NRMSE	R^2^
**Step-1**	CFN	71.6639	34.0019	39.0476	0.7730	51.2011	18.0998	18.8917	0.9054	66.3347	27.3379	25.4493	0.8504	30.9985	39.9996	51.5247	0.6935
	CAFN	78.0193	30.0238	39.2566	0.7706	51.6431	14.8440	19.2830	0.9014	69.3328	27.2765	27.0005	0.8316	28.6293	35.7132	50.7956	0.7021
	SGFN	75.9051	30.7651	38.7778	0.7761	49.9675	18.9392	19.0878	0.9034	61.3115	**23.9968**	25.8853	0.8453	28.9735	36.4945	53.6462	0.6677
	TCAFN	82.6223	33.2464	43.3819	0.7198	57.2008	**18.0841**	19.4565	0.8996	61.0273	26.3463	26.6528	0.8359	32.1624	42.2109	54.3580	0.6588
	**TSGAN**	**64.3873**	**28.5700**	**35.3055**	**0.8144**	**41.2731**	18.5214	**16.0634**	**0.9316**	**50.6590**	24.0737	**21.8624**	**0.8896**	**22.5640**	**30.5794**	**40.9926**	**0.8060**
**Step-2**	CFN	99.1526	39.4039	47.9565	0.6524	57.6752	20.7868	20.7699	0.8825	80.6233	29.6104	31.2263	0.7701	36.0391	**42.1946**	61.6295	0.5565
	CAFN	92.0269	37.0993	46.0879	0.6790	72.3334	22.3640	22.5438	0.8615	85.9460	32.3834	32.5420	0.7503	36.9187	51.6346	60.5104	0.5724
	SGFN	92.5065	36.5517	46.1352	0.6783	58.4521	19.0006	20.2438	0.8883	83.1260	29.2236	32.1480	0.7563	38.3016	47.7358	61.4073	0.5597
	TCAFN	96.0283	40.6010	44.2911	0.7035	68.7358	22.1131	22.5721	0.8612	90.9206	33.7150	33.6738	0.7326	44.1817	65.6499	67.0288	0.4754
	**TSGAN**	**84.8537**	**33.2236**	**41.6684**	**0.7376**	**55.6634**	**18.7006**	**19.7843**	**0.8934**	**68.8343**	**26.3776**	**28.4239**	**0.8095**	**29.8456**	43.7136	**51.9286**	**0.6851**
**Step-3**	CFN	108.8719	41.6778	49.2149	0.6278	66.1468	20.3830	23.0874	0.8512	115.5485	37.9933	36.9654	0.6717	44.9008	53.7312	74.8436	0.3407
	CAFN	101.6373	44.3786	46.8532	0.6627	69.8948	25.1153	22.7122	0.8560	94.4659	31.0804	34.4524	0.7149	43.3809	**52.4514**	71.3669	0.4006
	SGFN	110.8903	44.0573	48.5856	0.6373	65.9709	22.5818	23.0109	0.8522	103.7046	36.7228	35.7066	0.6937	46.8732	55.0313	72.8486	0.3754
	TCAFN	110.0806	46.6089	49.0675	0.6301	77.3831	24.7626	23.5015	0.8458	93.4088	31.5455	36.0346	0.6881	44.2866	54.2886	71.8448	0.3925
	**TSGAN**	**91.0199**	**34.1640**	**46.4626**	**0.6683**	**62.7747**	**19.7667**	**20.9817**	**0.8771**	**74.2571**	**28.0460**	**31.5499**	**0.7609**	**40.6783**	54.2884	**62.8839**	**0.5346**

**Note:** CFN represents the concatenation fusion method; CAFN represents the Cross-Attention fusion method; SGFN represents the selective gated fusion method; and TCAFN represents the Transformer-based Cross-Attention fusion method.

Overall, considering the results from both datasets, compared with the average performance of the other fusion methods, the proposed method reduced MAE, NRMSE, and SMAPE by 17.92%, 13.15%, and 13.31%, respectively, while increasing the average $R^{\text{2}}$ by 0.0472. Even compared with the best-performing fusion baselines, the proposed method achieved average improvements of 15.66%, 10.34%, 11.71%, and 9.46% in MAE, SMAPE, NRMSE, and $R^{\text{2}}$, respectively.

Fig 4 compares the MAE of different multimodal fusion strategies across four quarters and three forecasting horizons on the Albuquerque dataset. The bars represent the average error of each method at different forecasting horizons, the error bars denote approximate 95% confidence intervals calculated as ±1.96 times the standard error of the daily MAE values, and the dashed line represents the average performance improvement of TSGAN relative to the corresponding comparison fusion methods. As shown in the figure, compared with the single-modality methods, all multimodal fusion strategies achieved some degree of performance improvement, indicating clear complementarity between image and numerical information. However, significant differences remain among the fusion methods, indicating that multimodal forecasting performance depends not only on the incorporation of multiple information sources but also on how information is exchanged between modalities. Compared with the other fusion strategies, the proposed method maintains lower error levels across all four quarters and forecasting horizons, while its error bars are generally narrower. This suggests that the method not only improves forecasting accuracy but also reduces performance fluctuations across different scenarios.

**Fig 4:**
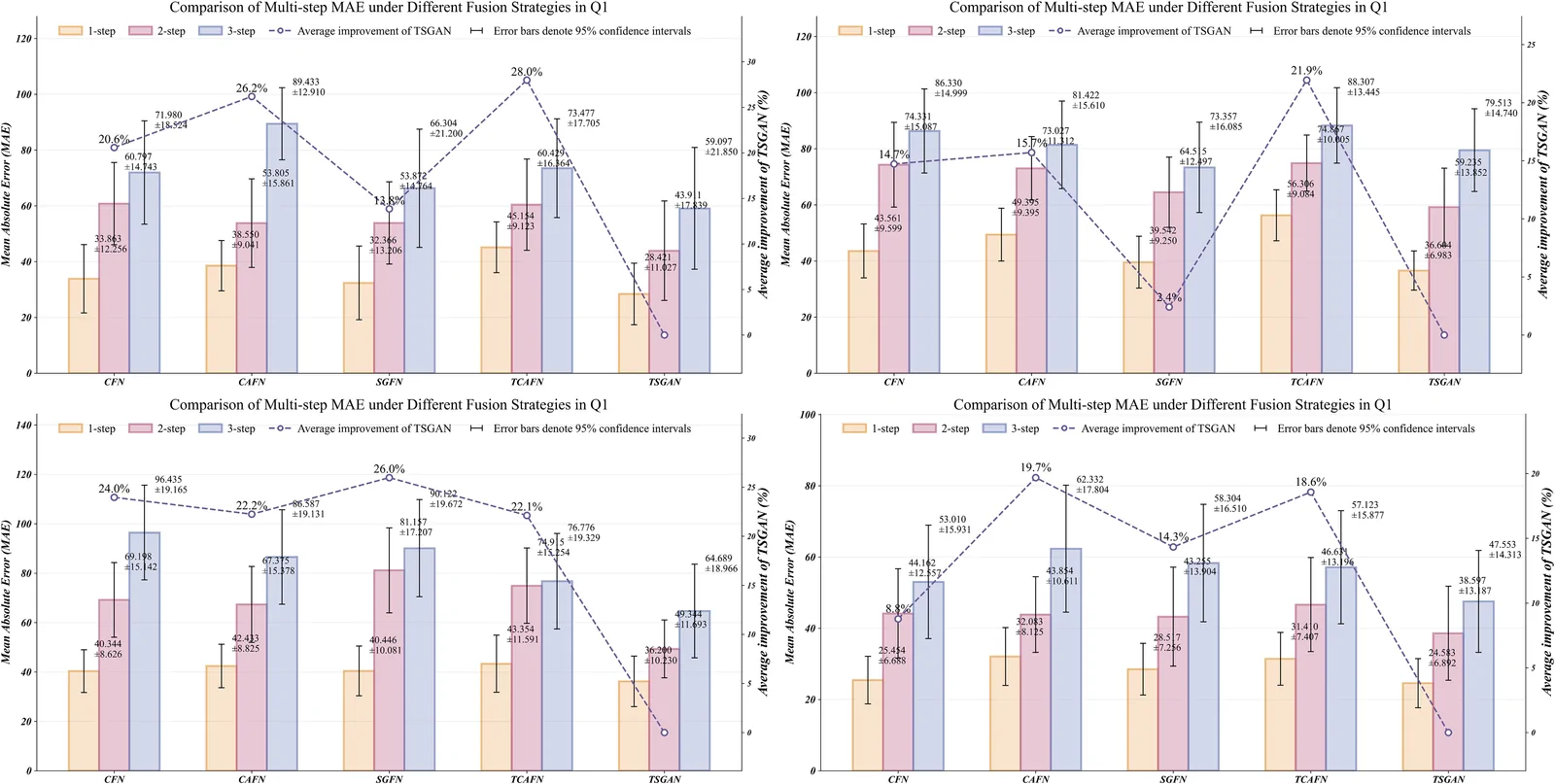
Prediction performance comparison under different fusion strategies. The bars represent the MAE values at different forecasting horizons, and the error bars denote approximate 95% confidence intervals calculated from the daily MAE values. The dashed line represents the average performance improvement of TSGAN relative to the comparison fusion methods.

Analysis of error variations across different fusion strategies reveals that, although some methods can reduce the average forecasting error by combining image and numerical information, they still exhibit significant fluctuations across different quarters and forecasting horizons. For example, certain fusion strategies achieve good forecasting results at Step 1, but as the forecasting horizon increases, their errors grow markedly, indicating that fixed fusion methods struggle to sustain effective information utilization over longer forecasting horizons. In contrast, the proposed method maintains a low error level even for Step 3 forecasts, with the average $R^{\text{2}}$ remaining high, indicating that this method can effectively mitigate error accumulation in longer-horizon forecasts. This is primarily because the proposed fusion strategy does not simply concatenate features from different modalities; rather, it adjusts the contributions of different types of information based on the current input state, enabling the model to focus more on effective features associated with current trends during the forecasting process.

Furthermore, the error bars of the proposed method are generally narrow, indicating greater stability across different quarters and weather conditions. Compared with fusion methods that exhibit significant increases in error under complex scenarios, the proposed method reduces the overall MAE by approximately 17.92% on average across the two datasets, while also exhibiting less performance variation across different experimental settings. This suggests that the dynamic fusion mechanism can reduce information bias caused by a single fixed fusion approach, enabling the model to maintain more consistent forecasting capabilities under different environmental conditions. Particularly under complex irradiance variations, the model prevents any single type of information from excessively dominating the forecasting process, thereby reducing forecasting bias in anomalous scenarios. Traditional multimodal fusion methods typically assume that different modalities have fixed levels of importance across all scenarios; however, in actual forecasting, the reliability of information from different sources varies with weather conditions and temporal scales. When the input information is highly relevant to the current state, a fixed fusion method can yield good forecasting results; however, when the validity of certain information decreases, excessive fusion may introduce additional interference. The proposed fusion strategy dynamically adjusts the information-interaction relationships between modalities, enabling the model to utilize features from different sources more flexibly and thereby enhancing the effectiveness of the joint representation.

Consequently, the experimental results demonstrate that improvements in multimodal forecasting performance stem not only from the joint input of image and numerical information but, more importantly, from effective synergy between the different modalities. Through dynamic information organization, the proposed method reduces forecasting errors while enhancing model stability across different quarters and forecasting horizons, thereby validating the effectiveness of the proposed fusion strategy in complex irradiance forecasting tasks.

## 4. Discussion

### 4.1 *Seasonal variation analysis*

Solar irradiance forecasting performance is influenced not only by model structure but also by the combined effects of seasonal variations in the Sun’s motion patterns and changes in cloud-evolution characteristics. The experimental results across the four quarters show that different models exhibit significant performance differences during seasonal transitions, indicating that irradiance time series exhibit strong seasonal dependence. Their variation patterns are not determined entirely by historical trends but are dynamically modulated by external environmental conditions. Although both datasets exhibit clear seasonal variations, the quarter-wise performance distributions are not completely consistent between the Albuquerque and Eugene sites, reflecting differences in local irradiance conditions and cloud-evolution characteristics. In general, when irradiance variations are relatively stable, historical irradiance time series can provide relatively reliable trend information, allowing models that rely partly on time-series characteristics to maintain a certain level of forecasting capability. Accordingly, TSGAN’s modality-selection mechanism can increase the contribution of historical time-series features. When irradiance becomes more strongly affected by cloud formation, movement, and dissipation, the time series exhibits stronger nonstationarity and short-term fluctuations. Under these conditions, models that rely solely on historical data struggle to promptly detect future changes in environmental conditions and are prone to interpreting sudden decreases or recoveries in irradiance as anomalous disturbances, thereby leading to forecasting lags and error accumulation. Fig 5 illustrates the variation in forecasting performance across different quarters on the Albuquerque dataset.

**Fig 5:**
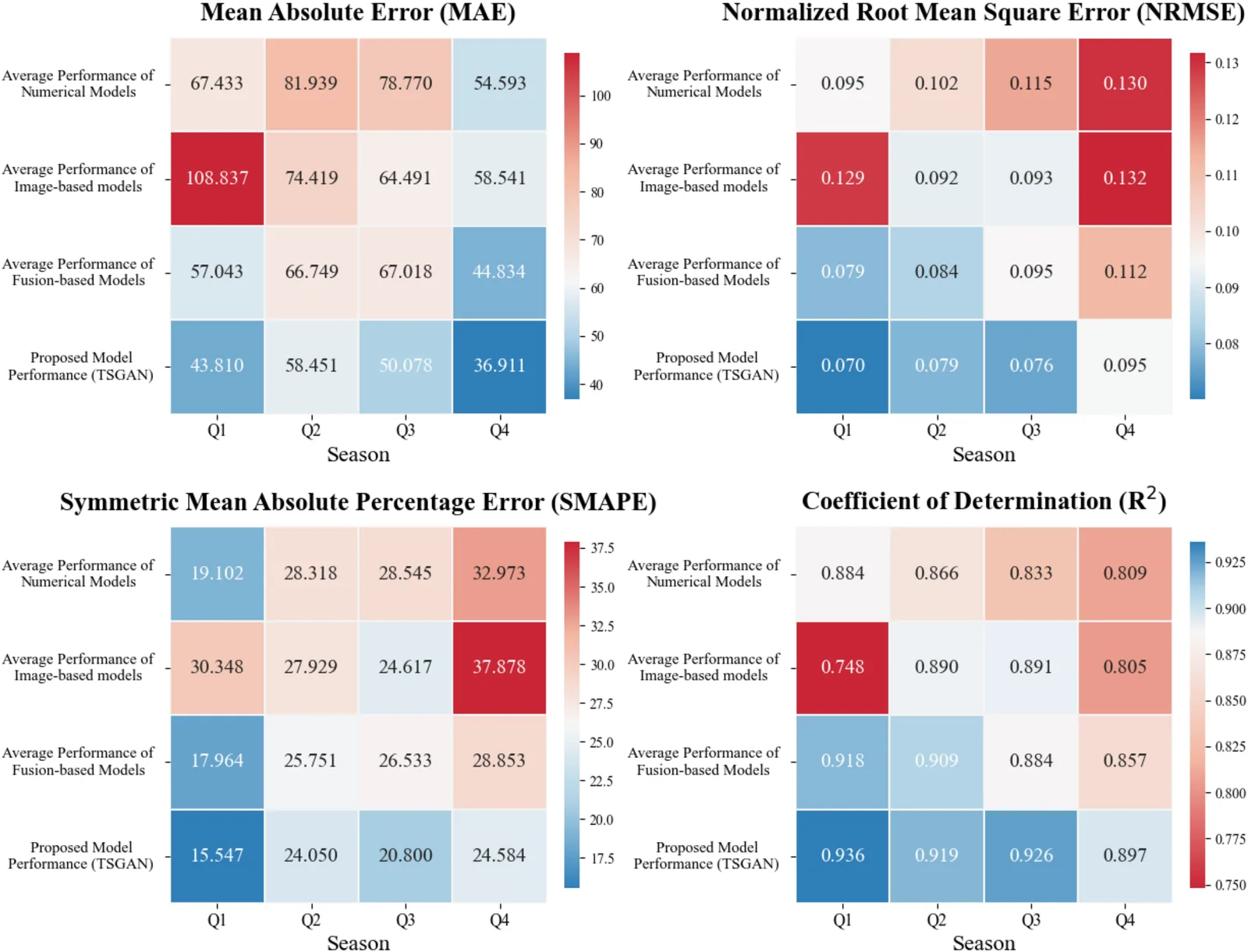
Comparison of the proposed model with different models under seasonal conditions.

As shown in Fig 5, seasonal variations have a relatively significant impact on the forecasting performance of different types of models; however, the proposed TSGAN consistently maintains optimal or near-optimal performance across all four quarters. Specifically, whether evaluated using error metrics such as MAE, NRMSE, and SMAPE or the goodness-of-fit metric $R^{\text{2}}$, TSGAN outperforms the numerical, image-based, and general multimodal fusion methods. At the same time, TSGAN exhibits relatively small performance fluctuations across different quarters; for example, its NRMSE consistently remains low, while its $R^{\text{2}}$ remains high. This indicates that although seasonal variations alter irradiance levels and weather conditions, they do not significantly diminish the model’s relative advantage. In contrast, the single-image model exhibits significant fluctuations in Q1 and Q4, indicating that the predictive validity of sky images is easily affected by changes in the Sun’s altitude and seasonal cloud conditions. Although the single-numerical-modality model is generally more stable, its error gradually increases in Q2, Q3, and Q4, indicating that when relying solely on historical irradiance and meteorological time series, the model’s adaptability to rapid cloud changes and seasonal distributions remains limited. General fusion methods can mitigate the information deficiencies of single-modality approaches to some extent, but their performance still fluctuates significantly with seasonal variations, indicating that simple concatenation or fixed fusion cannot consistently ensure the effective use of information from different modalities.

TSGAN demonstrates greater stability across different seasons, primarily because its modality-selection mechanism dynamically adjusts the contributions of the two modalities based on current weather conditions and irradiance changes. When irradiance variations are relatively stable, the model can rely more heavily on historical numerical sequences to maintain the irradiance baseline and continuity of variation; when rapid irradiance fluctuations occur because of cloud movement or dissipation, the model can enhance the contribution of cloud-structure and spatial-variation information from sky images. Through this state-dependent modality filtering, the model reduces interference from lagging numerical information and conflicts between modalities in the joint representation, enabling multisource information to achieve more effective complementarity and coupling under different seasonal conditions. Therefore, TSGAN’s performance improvement does not stem solely from an increase in the amount of input information but more likely from its ability to dynamically identify the reliability of different modalities under seasonal changes and maintain more stable and effective information synergy under complex weather conditions.

### 4.2 DM test results

To further verify the statistical reliability of the performance improvements observed above, this paper employs the Diebold-Mariano (DM) test to conduct pairwise comparisons of forecasting errors between TSGAN and the main comparison models under different prediction horizons. The absolute error is used as the loss function. To account for the serial dependence associated with overlapping multi-step forecasts, the long-run variance is estimated using the Newey–West HAC estimator with Bartlett weights. Holm’s step-down correction is applied separately within each dataset to address multiple comparisons, and the results are presented in Tables 9 and 10.

**Table 10:** DM test results of the proposed model compared with benchmark models on the Eugene dataset.

Model	Q1			Q2			Q3			Q4		
	1-step	2-step	3-step	1-step	2-step	3-step	1-step	2-step	3-step	1-step	2-step	3-step
MLP	−5.9988^***^	−4.8565^***^	−6.3479^***^	−10.0735^***^	−9.6930^***^	−7.3731^***^	−7.7539^***^	−4.6150^***^	−6.9875^***^	−6.4115^***^	−4.8117^***^	−2.7425^*^
GRU	−4.7191^***^	−4.8590^***^	−4.4799^***^	−8.0111^***^	−6.4609^***^	−4.8629^***^	−6.3052^***^	−3.6431^***^	−6.1978^***^	−5.9471^***^	−4.4106^***^	−1.4975
LSTM	−5.8612^***^	−3.6778^***^	−3.8486^***^	−10.2526^***^	−7.4631^***^	−6.6383^***^	−4.4666^***^	−4.6783^***^	−5.2467^***^	−5.2176^***^	−4.7263^***^	−1.6152
RF	−9.2521^***^	−8.1216^***^	−8.4491^***^	−2.7132^*^	−1.6670	−1.3567	−1.1815	−0.1105	−1.8585	−4.8316^***^	−4.7888^***^	−1.8886
XGBoost	−12.4016^***^	−10.7158^***^	−10.0190^***^	−14.3246^***^	−10.7449^***^	−10.2688^***^	−8.0606^***^	−4.0125^***^	−4.4210^***^	−8.7135^***^	−7.7591^***^	−4.2992^***^
Transformer	−9.6070^***^	−8.0738^***^	−7.6901^***^	−5.9792^***^	−4.1641^***^	−4.4577^***^	−2.2739	−0.3745	−1.3799	−4.5839^***^	−4.6202^***^	−1.7896
PatchTST	−7.7108^***^	−5.7795^***^	−4.3507^***^	−12.4413^***^	−7.3598^***^	−9.9716^***^	−7.2096^***^	−4.3498^***^	−8.2679^***^	−6.3817^***^	−4.8200^***^	−2.2226
CNN	−5.4919^***^	−4.4940^***^	−3.0096^**^	−8.6640^***^	−14.5637^***^	−7.1528^***^	−8.1501^***^	−5.1298^***^	−6.4300^***^	−5.8805^***^	−5.1395^***^	−2.3784
CNN-LSTM	−5.4521^***^	−3.7554^***^	−4.0269^***^	−12.5589^***^	−12.4161^***^	−7.9686^***^	−13.6478^***^	−4.1709^***^	−4.5894^***^	−13.0071^***^	−9.2558^***^	−5.0885^***^
ResNet-LSTM	−7.2136^***^	−4.9320^***^	−4.2255^***^	−13.0726^***^	−4.7709^***^	−3.1953^**^	−12.9942^***^	−5.4107^***^	−4.7458^***^	−13.1905^***^	−11.1825^***^	−9.7351^***^
CNN-GRU	−10.7080^***^	−8.2331^***^	−7.0654^***^	−13.8411^***^	−3.3274^**^	−4.2695^***^	−15.3137^***^	−5.7297^***^	−5.8492^***^	−11.1723^***^	−8.9760^***^	−3.8732^***^
ResNet-GRU	−8.2913^***^	−12.4910^***^	−3.8319^***^	−11.6583^***^	−5.3854^***^	−5.3388^***^	−14.4763^***^	−5.4978^***^	−7.2788^***^	−12.6918^***^	−9.6828^***^	−4.9004^***^
CFN	−3.2200^**^	−5.1559^***^	−5.3342^***^	−6.4125^***^	−1.3395	−1.4014	−7.5848^***^	−3.3227^**^	−12.0828^***^	−9.0961^***^	−4.2942^***^	−1.9336
CAFN	−6.2898^***^	−2.5295	−2.7599^*^	−6.5816^***^	−8.2303^***^	−2.8254^*^	−8.3725^***^	−4.8573^***^	−5.6744^***^	−6.4719^***^	−5.1146^***^	−1.3815
SGFN	−5.2244^***^	−2.7721^*^	−6.0869^***^	−5.6935^***^	−1.8251	−1.3605	−4.9558^***^	−3.9331^***^	−8.5068^***^	−5.1459^***^	−5.3344^***^	−2.5599
TCAFN	−6.9222^***^	−4.5834^***^	−5.5450^***^	−10.3730^***^	−6.1943^***^	−6.0329^***^	−4.3963^***^	−6.0901^***^	−4.8823^***^	−8.4819^***^	−8.3446^***^	−1.7821

**Note:** ^***^, ^**^, and ^*^ indicate significance at the 1%, 5%, and 10% levels, respectively. The DM statistic is calculated based on absolute error loss and uses a one-tailed test; a negative value indicates that TSGAN has a lower prediction loss than the corresponding comparison model.

**Table 9:** DM test results of the proposed model compared with benchmark models on the Albuquerque dataset.

Model	Q1			Q2			Q3			Q4		
	1-step	2-step	3-step	1-step	2-step	3-step	1-step	2-step	3-step	1-step	2-step	3-step
MLP	−12.7788^***^	−11.7851^***^	−10.0271^***^	−9.3577^***^	−10.5347^***^	−8.0600^***^	−18.8208^***^	−12.5396^***^	−11.4990^***^	−10.6755^***^	−8.1341^***^	−6.7784^***^
GRU	−6.4584^***^	−6.5432^***^	−5.0078^***^	−8.5068^***^	−8.3165^***^	−6.3110^***^	−15.5039^***^	−9.2549^***^	−9.1397^***^	−8.8954^***^	−5.5422^***^	−8.4642^***^
LSTM	−10.4720^***^	−10.9580^***^	−7.0116^***^	−8.4164^***^	−9.1363^***^	−9.1489^***^	−11.6417^***^	−12.8344^***^	−10.6713^***^	−11.0516^***^	−9.0427^***^	−6.8990^***^
RF	−11.8516^***^	−8.6125^***^	−8.1876^***^	−9.3329^***^	−9.3748^***^	−8.6126^***^	−7.4249^***^	−8.1984^***^	−6.9713^***^	−7.9269^***^	−8.1162^***^	−7.3516^***^
XGBoost	−19.0204^***^	−13.8000^***^	−10.6880^***^	−19.7737^***^	−15.1655^***^	−12.7007^***^	−21.4039^***^	−15.0065^***^	−11.9309^***^	−21.6267^***^	−15.1313^***^	−13.6388^***^
Transformer	−14.1962^***^	−9.9908^***^	−9.2250^***^	−12.2544^***^	−9.5931^***^	−9.1045^***^	−10.3229^***^	−10.7285^***^	−8.4087^***^	−10.6161^***^	−8.4612^***^	−8.5239^***^
PatchTST	−10.2675^***^	−8.7142^***^	−7.1272^***^	−7.9009^***^	−5.6173^***^	−5.3294^***^	−13.7082^***^	−13.7975^***^	−8.7120^***^	−12.7152^***^	−6.0573^***^	−7.0449^***^
CNN	−9.1898^***^	−6.7949^***^	−5.7098^***^	−8.2501^***^	−7.7571^***^	−1.6850	−9.0908^***^	−9.8981^***^	−11.3183^***^	−8.4704^***^	−10.1418^***^	−6.9812^***^
CNN-LSTM	−17.6320^***^	−23.5472^***^	−10.0034^***^	−6.5151^***^	−5.9130^***^	−3.5773^***^	−3.0690^**^	−3.5580^***^	−3.1681^**^	−9.8048^***^	−6.3174^***^	−10.9104^***^
ResNet-LSTM	−17.8175^***^	−24.2792^***^	−12.3518^***^	−9.8929^***^	−7.5286^***^	−2.5580^**^	−10.1358^***^	−8.3019^***^	−7.8416^***^	−27.6246^***^	−7.8710^***^	−7.3029^***^
CNN-GRU	−18.9347^***^	−13.4203^***^	−8.1658^***^	−9.1431^***^	−6.6870^***^	−6.0934^***^	−5.7215^***^	−5.4476^***^	−3.4185^***^	−10.3610^***^	−5.9171^***^	−8.7862^***^
ResNet-GRU	−24.9044^***^	−16.8316^***^	−8.4329^***^	−6.4990^***^	−5.6567^***^	−5.4960^***^	−9.2932^***^	−8.8533^***^	−6.3484^***^	−19.8362^***^	−5.8865^***^	−7.9785^***^
CFN	−4.2220^***^	−8.4955^***^	−4.9562^***^	−4.9931^***^	−6.5308^***^	−2.5532^**^	−4.6039^***^	−8.5471^***^	−11.0671^***^	−0.9781	−3.2117^***^	−2.1690^*^
CAFN	−7.6372^***^	−4.4062^***^	−8.5703^***^	−8.4485^***^	−6.0782^***^	−0.6136	−5.2414^***^	−7.8175^***^	−7.9000^***^	−7.0235^***^	−2.9125^**^	−6.8276^***^
SGFN	−2.9367^**^	−4.3878^***^	−3.5406^***^	−2.2395^*^	−2.6934^**^	2.4160	−3.7449^***^	−13.9326^***^	−8.8966^***^	−3.7641^***^	−2.7014^**^	−4.6332^***^
TCAFN	−11.1843^***^	−6.4616^***^	−5.6044^***^	−13.3398^***^	−7.0923^***^	−3.3013^***^	−6.2304^***^	−10.9694^***^	−4.7956^***^	−7.1169^***^	−4.7417^***^	−4.2579^***^

**Note:** ^***^, ^**^, and ^*^ indicate significance at the 1%, 5%, and 10% levels, respectively. The DM statistic is calculated based on absolute error loss and uses a one-tailed test; a negative value indicates that TSGAN has a lower prediction loss than the corresponding comparison model.

The test results show that, across both datasets, 92.97% and 91.15% of the comparisons remain statistically significant at the 10% and 5% levels, respectively, after Holm correction. In addition, nearly all DM statistics are negative, indicating that TSGAN achieves lower mean forecasting loss than the corresponding comparison models in the vast majority of cases. These results provide statistical evidence supporting TSGAN’s forecasting improvements after accounting for multiple comparisons. Furthermore, statistically significant improvements are observed in both datasets across different quarters and forecasting horizons, supporting the model’s relative advantage under different seasonal conditions and forecasting lead times. Therefore, the DM test results further support the preceding experimental conclusions from a statistical inference perspective and validate the effectiveness of the proposed modality-selective attention-gating mechanism in coordinating image information with numerical states and enhancing forecasting stability and reliability.

### 4.3 Sensitivity analysis of key hyperparameters

To evaluate the sensitivity of the model to key hyperparameters, a one-factor-at-a-time strategy was adopted. Specifically, the shared hidden representation dimension ($d_{model}$), which determines the dimensionality of the numerical and visual features processed by the Transformer encoders and multimodal fusion module, together with the learning rate (η) and dropout rate (p), was varied individually. The experiments were conducted on one-step forecasting tasks for the four quarters of the Albuquerque and Eugene datasets. The baseline hyperparameter setting was ($d_{model}$=128), (η=$10^{-4}$), and (p=0.05).

As shown in Table 11, the overall performance of the model exhibited only limited variation under different hyperparameter settings, with NRMSE and $R^{\text{2}}$ showing particularly high stability. On the Albuquerque dataset, the four-quarter average NRMSE under different parameter settings ranged from 12.2680% to 12.9990%, corresponding to a maximum variation of only 0.731 percentage points. The average $R^{\text{2}}$ ranged from 0.9600 to 0.9648, with an absolute variation of no more than 0.0048. On the Eugene dataset, the average NRMSE ranged from 28.5560% to 30.0240%, while the average $R^{\text{2}}$ ranged from 0.8459 to 0.8604. Compared with the baseline configuration, the average MAE under the other parameter settings varied by approximately 9.20% and 8.84% on the Albuquerque and Eugene datasets, respectively. Although different hyperparameter settings led to certain performance fluctuations, no obvious performance degradation or substantial loss of predictive capability was observed, indicating that the model is relatively robust to the key hyperparameters.

**Table 11:** Sensitivity analysis of key hyperparameters for TSGAN on different datasets.

Dataset	Model	Q1				Q2				Q3				Q4			
		MAE	SMAPE	NRMSE	R^2^	MAE	SMAPE	NRMSE	R^2^	MAE	SMAPE	NRMSE	R^2^	MAE	SMAPE	NRMSE	R^2^
**Albuquerque**	Baseline	**28.4211**	10.7657	9.5866	0.9708	36.6041	20.1119	12.6762	0.9674	36.1998	**14.6895**	12.3918	0.9609	**24.5830**	**17.4334**	15.2121	0.9552
	$d_{model}$=64	33.0817	11.0413	9.2722	0.9727	39.3426	15.6622	13.2063	0.9646	42.7883	21.1454	12.8564	0.9580	27.0102	19.4581	15.5657	0.9531
	$d_{model}$=256	31.2534	10.4210	9.2441	0.9729	**34.7171**	**14.3756**	**12.2341**	**0.9697**	42.0401	17.9256	13.2941	0.9550	29.6676	22.9879	17.2223	0.9425
	p=0	30.7586	**9.9110**	9.3839	0.9721	37.3026	16.2263	13.5697	0.9627	**35.0485**	15.1047	11.8152	0.9645	28.9791	22.3735	16.8702	0.9449
	p=0.1	29.6481	10.8828	**8.9587**	**0.9745**	37.3912	15.7441	12.8475	0.9665	36.1613	16.8925	**11.7835**	**0.9647**	26.0960	18.9444	16.2865	0.9486
	η=10 ^-3^	32.6483	12.6800	9.0782	0.9738	40.3037	17.6143	12.7277	0.9672	40.9784	19.9931	12.2419	0.9619	29.4586	20.6606	16.9638	0.9443
	η=10 ^-5^	32.5424	11.8785	9.1550	0.9734	40.5933	18.6379	12.6707	0.9675	38.6188	18.4884	12.3744	0.9611	27.8346	22.9875	**14.8722**	**0.9572**
**Eugene**	Baseline	64.3873	28.5700	35.3055	0.8144	**41.2731**	18.5214	**16.0634**	**0.9316**	**50.6590**	24.0737	**21.8624**	**0.8896**	**22.5640**	30.5794	40.9926	0.8060
	$d_{model}$=64	67.3067	**26.6046**	35.1977	0.8156	47.0544	15.4240	16.9156	0.9241	53.9344	21.6450	22.6205	0.8818	25.9376	47.4488	43.0380	0.7861
	$d_{model}$=256	71.5897	29.3463	36.2631	0.8042	49.7192	19.4609	17.0699	0.9227	54.3865	25.5024	22.8805	0.8791	23.8898	31.2335	43.8808	0.7777
	p=0	73.2332	30.4071	37.2885	0.7930	46.0205	19.6216	16.8580	0.9246	51.3575	**19.8314**	22.1909	0.8863	23.7213	30.8008	41.7733	0.7985
	p=0.1	68.5158	29.2280	35.2756	0.8147	46.2735	20.5619	16.6926	0.9261	54.7439	25.4557	22.2279	0.8859	22.8836	**30.4354**	**40.4565**	**0.8110**
	η=10 ^-3^	75.1185	30.8905	36.6905	0.7996	46.7250	**14.9263**	17.6696	0.9172	55.2900	24.5999	21.8771	0.8895	25.2726	35.7817	42.9986	0.7865
	η=10 ^-5^	**66.6269**	27.5191	**34.8312**	**0.8194**	41.5631	16.5514	17.2375	0.9212	51.3298	22.3222	22.3829	0.8843	25.6630	39.9899	43.8355	0.7781

Considering the results across different datasets, quarters, and evaluation metrics, the baseline configuration $d_{model}$=128, η=$10^{-4}$, and p=0.05 provided the most balanced overall performance. When the results were averaged across the Albuquerque and Eugene datasets, the baseline configuration achieved the best MAE, NRMSE, and $R^{\text{2}}$, with values of 38.0864, 20.5113%, and 0.9120, respectively. Although p=0 yielded a slightly lower average SMAPE of 20.5345%, compared with 20.5931% for the baseline configuration, the difference was only 0.0586 percentage points. Therefore, these results support the use of the baseline configuration as the default hyperparameter setting adopted in the main experiments.

## 5. Conclusion and future work

### 5.1 Conclusion

This paper proposes a multimodal adaptive fusion framework for ultra-short-term solar irradiance forecasting. By jointly modeling historical irradiance time series and dynamic cloud information from sky images, the framework effectively overcomes the limitations of traditional single-source forecasting methods, which struggle to capture rapid fluctuations caused by local cloud changes under complex weather conditions. Rather than simply adding input modalities, this method establishes deep correlations between numerical state evolution and changes in the visual environment, enabling the model to simultaneously learn historical patterns of irradiance variation and potential future perturbations, thereby enhancing forecasting accuracy, stability, and adaptability to complex scenarios. Based on systematic experimental validation across different quarters, forecasting horizons, and multiple benchmark models, this paper draws the following main conclusions:

(1) The unified state encoder proposed in this paper effectively addresses the limited ability of traditional numerical baseline models to detect abrupt changes induced by localized clouds. Experimental results show that although models relying solely on historical irradiance and meteorological variables can effectively learn the periodic variation patterns of solar irradiance, they depend primarily on past state information and lack the ability to anticipate sudden irradiance changes caused by factors such as cloud cover and rapid cloud movement. Consequently, when the forecasting horizon is extended or weather conditions become more complex, a single numerical-modality model is prone to forecasting lags and cumulative errors. In contrast, by incorporating additional visual information about the sky, the proposed method enables the model not only to capture temporal dependencies in irradiance sequences but also to utilize external environmental states to anticipate future changes, thereby addressing, at the mechanistic level, the lack of leading information in traditional time-series models.
(2) The multimodal joint representation method proposed in this paper effectively addresses the limitations of single-image models in anchoring absolute irradiance levels and maintaining historical continuity. Although visual models such as CNNs and ResNets can extract dynamic features—such as solar occlusion and cloud movement—from sky images, they struggle to establish a stable mapping between visual states and absolute irradiance levels when relying solely on image information. As a result, their forecasts are susceptible to variations in the Sun’s position and image quality. Experimental results indicate that visual features alone cannot fully address the irradiance forecasting problem. Images primarily describe the current state of the sky but lack constraints from the historical evolution of irradiance and patterns of solar motion, potentially leading to inconsistencies between visual states and actual irradiance responses. The proposed model integrates the historical physical patterns provided by the numerical modality with the prior information on future changes provided by the image modality. This transforms the forecasting process from simple state matching into comprehensive modeling of irradiance variation mechanisms, thereby effectively reducing the forecasting bias caused by a single visual representation.
(3) The state-aware adaptive fusion mechanism proposed in this paper effectively overcomes the limitation of traditional fusion mechanisms in responding to changes in modality reliability. Existing multimodal methods typically employ feature concatenation, fixed-weight fusion, or shallow interaction approaches. Although these methods can simultaneously utilize information from multiple sources, they assume that the relative importance of different modalities remains fixed across weather conditions, making it difficult to adapt to dynamic changes in modality reliability during actual forecasting. Simple fusion methods struggle to fully exploit the coupling relationships between modalities, whereas an effective cross-modal interaction mechanism is essential for improving forecasting performance. The proposed TSGAN further transforms the fusion process into a state-driven information-filtering process, enabling the model to dynamically adjust the contributions of the numerical and image modalities based on current irradiance changes. Under clear-sky or stable conditions, the model can make greater use of continuity information from historical time series, whereas under rapidly changing cloud cover or local shading conditions, it enhances the contribution of visual information, thereby reducing the information bias caused by fixed fusion strategies. Experimental results show that, compared with other multimodal fusion methods, the proposed method maintains lower error levels across all four quarters and different forecasting horizons while exhibiting narrower error ranges, indicating that this mechanism not only improves forecasting accuracy but also enhances cross-scenario stability.

### 5.2 Future work

The TSGAN proposed in this paper offers a forecasting approach that shifts from “information superposition” to “state selection” for the collaborative modeling of ground-based sky images and historical numerical time series. It can provide more timely irradiance information for photovoltaic power smoothing, energy-storage control, and short-term grid dispatch. The current findings remain limited by the scope of the data and the forecasting horizons. In addition, the present framework focuses on deterministic point forecasting and does not explicitly characterize the uncertainty associated with future irradiance variations. Recent studies have demonstrated that deep stochastic probabilistic forecasting frameworks can provide useful uncertainty information for short-term solar power forecasting [44]. Future research could incorporate continuous cloud-motion characterization and satellite or infrared imagery and evaluate the model’s applicability under distribution shifts and uncertainty quantification through cross-site validation and probabilistic interval forecasting.
